# Analyzing the research landscape: Mapping frontiers and hot spots in anti-cancer research using bibliometric analysis and research network pharmacology

**DOI:** 10.3389/fphar.2023.1256188

**Published:** 2023-09-07

**Authors:** Qi Han, Zhongxun Li, Yang Fu, Hongliang Liu, Huina Guo, Xiaoya Guan, Min Niu, Chunming Zhang

**Affiliations:** ^1^ Shanxi Key Laboratory of Otorhinolaryngology Head and Neck Cancer, First Hospital of Shanxi Medical University, Taiyuan, China; ^2^ Shanxi Province Clinical Medical Research Center for Precision Medicine of Head and Neck Cancer, First Hospital of Shanxi Medical University, Taiyuan, China; ^3^ Department of Cardiology, Shanxi Cardiovascular Hospital, Taiyuan, China; ^4^ Department of Otolaryngology Head and Neck Surgery, First Hospital of Shanxi Medical University, Taiyuan, China; ^5^ Department of Cell Biology and Genetics, The Basic Medical School of Shanxi Medical University, Taiyuan, Shanxi, China

**Keywords:** network pharmacology, anti-cancer, bibliometric analysis, citespace, research frontiers

## Abstract

**Introduction:** Network pharmacology has emerged as a forefront and hotspot in anti-cancer. Traditional anti-cancer drugs are limited by the paradigm of “one cancer, one target, one drug,” making it difficult to address the challenges of recurrence and drug resistance. However, the main advantage of network pharmacology lies in its approach from the perspective of molecular network relationships, employing a “one arrow, multiple targets” strategy, which provides a novel pathway for developing anti-cancer drugs. This study employed a bibliometric analysis method to examine network pharmacology’s application and research progress in cancer treatment from January 2008 to May 2023. This research will contribute to revealing its forefront and hotspots, offering new insights and methodologies for future investigations.

**Methods:** We conducted a literature search on network pharmacology research in anti-cancer (NPART) from January 2008 to May 2023, utilizing scientific databases such as Web of Science Core Collection (WoSCC) and PubMed to retrieve relevant research articles and reviews. Additionally, we employed visualization tools such as Citespace, SCImago Graphica, and VOSviewer to perform bibliometric analysis.

**Results:** This study encompassed 3,018 articles, with 2,210 articles from WoSCC and 808 from PubMed. Firstly, an analysis of the annual national publication trends and citation counts indicated that China and the United States are the primary contributing countries in this field. Secondly, the recent keyword analysis revealed emerging research hotspots in “tumor microenvironment,” “anti-cancer drugs,” and “traditional Chinese medicine (TCM). “ Furthermore, the literature clustering analysis demonstrated that “calycosin,” “molecular mechanism,” “molecular docking,” and “anti-cancer agents” were widely recognized research hotspots and forefront areas in 2023, garnering significant attention and citations in this field. Ultimately, we analyzed the application of NPART and the challenges.

**Conclusion:** This study represents the first comprehensive analysis paper based on bibliometric methods, aiming to investigate the forefront hotspots of network pharmacology in anti-cancer research. The findings of this study will facilitate researchers in swiftly comprehending the current research trends and forefront hotspots in the domain of network pharmacology in cancer research.

## Introduction

With the continuous development of technology, network pharmacology is becoming a frontier and hotspot in anti-cancer therapy. The advantage of network pharmacology lies in its ability to explain the basis of complex biological systems from a network perspective (Xin et al., 2021). Traditional drug discovery methods are dominated by the paradigm of “one disease, one target, one drug,” but this paradigm has multiple limitations. With the latest advances in systems biology, the focus of drug discovery has shifted from “single target” to “multi-target drugs,” which has important theoretical and practical significance (Jorgensen, 2011; Lu et al., 2012; Peter, 2020). As an interdisciplinary field, network pharmacology utilizes systems biology and computer science technologies to explore the molecular mechanisms and drug targets of complex diseases, which is of great significance for solving problems in anti-cancer research. Establishing molecular relationship networks can reveal the interactions between genes, drugs, and their targets in various diseases (Chen and Wu, 2013; Diaz-Beltran et al., 2013) and study how perturbations in cellular networks lead to human malignant tumors (Wei et al., 2021) and other phenotypes. In addition, network pharmacology supports the search for new therapeutic pathways by analyzing the complex network of molecular interactions to reveal the molecular mechanisms of cancer drug resistance. Potential anticancer compound brucine may overcome drug resistance in breast cancer (Qayoom et al., 2023). Moreover, network pharmacology has been widely used in recent years to elucidate the mechanism of action of traditional Chinese medicine (TCM) for cancer treatment and to screen the active ingredients and critical targets of TCM by constructing networks of compounds and targets (Wu et al., 2023). The study found that the Chinese medicine compound preparation Xihuangwan (Wu et al., 2020), Compound kushen injection (CKI) (Guo et al., 2023), and Calycosin (Hu et al., 2023) inhibit tumor growth by multi-targeting the genes related to breast, esophageal, and colon cancer, respectively. Therefore, it is essential to investigate cancer initiation and treatment mechanisms through network pharmacology.

In the past few decades, researchers have been devoted to finding drugs to treat cancer and have made tremendous progress in areas such as chemotherapy drugs (Wang Y. et al., 2023), targeted drugs (Isaksson et al., 2023; Jiang et al., 2023), immunotherapy (Ren et al., 2023; Xiong et al., 2023; Yadav et al., 2023), sonodynamic therapy (Wu et al., 2022), and radiation therapy (Eyre-Walker and Stoletzki, 2013; Dumago et al., 2023; Moon et al., 2023). However, these anti-cancer strategies have shortcomings, including side effects and cancer recurrence. Recent studies have shown that network pharmacology methods can help predict drug targets and side effects and improve treatment efficacy. In oncology, network pharmacology has been widely used in drug screening and development, drug repurposing, and the study of drug mechanisms. For example, by constructing interaction and signal transduction networks, potential drug targets can be predicted, and new therapeutic drugs can be discovered (Hao and Xiao, 2014; Wang et al., 2020; Yan et al., 2022). In addition, through the simulation of network pharmacology, drug safety and efficacy can be evaluated, and treatment plans can be optimized to improve personalized treatment outcomes.

This article is the first to systematically analyze the anti-cancer research field through bibliometric methods (Cooper, 2015). This study employs bibliometric methodologies to comprehensively analyze the application and research progress of network pharmacology in oncology treatment. It spans from January 2008 to May 2023. The investigation is conducted from five distinct perspectives: 1) Trends in national annual publication output; 2) Collaborative networks among countries and institutions; 3) Author collaboration networks and contributions; 4) Keyword co-occurrence and clustering analysis; and 5) Co-citation and clustering analysis of literature. Based on the outcomes of these analyses, we explore the current state and future directions of network pharmacology in cancer research. Furthermore, we discuss the applications and challenges of network pharmacology in cancer research. It is the aim of this study to provide novel perspectives and methodologies in the area of NPART to prospective researchers.

## Materials and methods

### Data collection and research strategy

Web of Science Core Collection (WoSCC) is a comprehensive academic database covering over 190 subject areas globally. It is widely recognized as the superior database for bibliometric research in various disciplines, providing excellent literature retrieval and citation analysis services (Cheng et al., 2022b; Zhang et al., 2022; 2023).

In contrast to WoSCC, the freely accessible PubMed database, curated by the United States National Library of Medicine, is updated daily. This database comprises over 34 million citations that are readily accessible *via* the internet (Cui et al., 2023). The PubMed MeSH (Medical Subject Headings) system uses standardized vocabulary for subject annotations and categorizations of medical literature (Medical Subject Headings Home Page, 2023). MeSH terms can be precisely identified and retrieved in literature databases using a unique identifier code. Consequently, both the WoSCC and PubMed databases provide excellent utility within this study.

**FIGURE 1:**
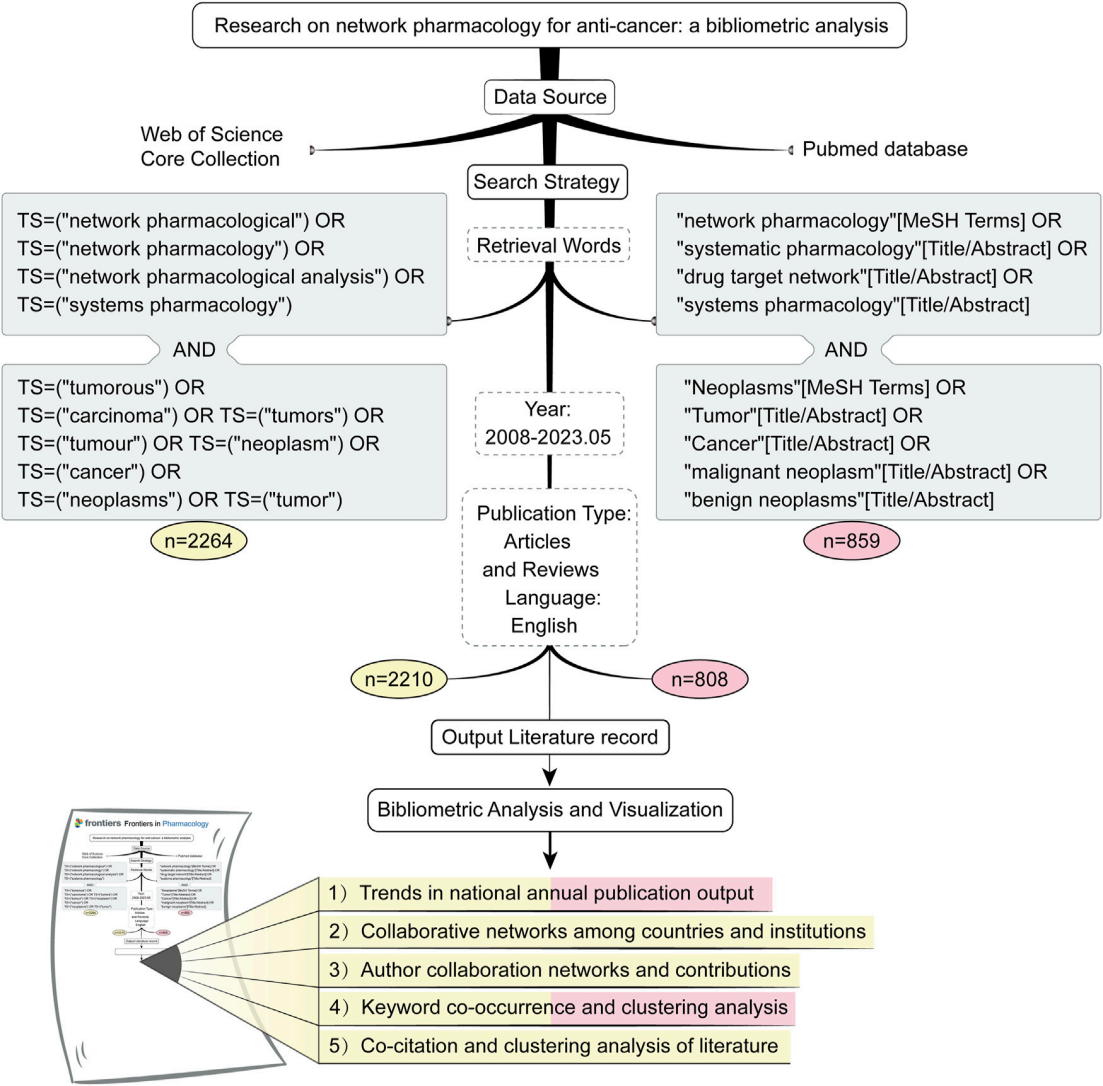
A flowchart of the literature strategy and data analysis.

We have provided a more comprehensive depiction of our elaborate search approach in Figure 1. 3,123 original articles were retrieved from the WoSCC database by topic searching (TS) and the PubMed database using MeSH and Title/abstract. Subsequently, 3,018 articles were screened based on publication year (2008–2023.05), publication type (articles and reviews), and language (English). Finally, the retrieved files were exported in the form of references.

We conducted distinct searches in the WoSCC and PubMed databases, meticulously removing duplicate papers, and proceeded with subsequent in-depth bibliometric analyses. To ensure the utmost objectivity and comprehensiveness of our research, we scrutinized the annual publication trends of NPART within these two databases. We further prognosticated forthcoming publication trends. Additionally, we provide accurate and comprehensive predictions of NPART research hotspots through keyword analysis on both databases. As for the remaining sections, we analyzed them separately using the WoSCC database.

### Grey prediction model

The grey prediction model GM(1,1) is a commonly used nonlinear prediction method suitable for short-term forecasting (Luo et al., 2020). The primary objective is to analyze the developmental trend of sequence data and establish a grey differential equation model to predict future trends. In this study, we utilized a standard GM(1,1) model implemented through R 4.3.0 software to forecast the annual publication volume of anti-cancer network pharmacology research for the next 4 years.

### Bibliometric analysis and visualization

The records of the retrieved publications were exported to BiblioMetrix, Citespace, and SCImago Graphica for further bibliometric analysis. The BiblioMetrix software package (R4.3.0) captured and extracted bibliographic information about the selected publications, including subjects, authors, keywords, and country distribution (Aria and Cuccurullo, 2017). The H-index measures scientific output and quantitatively assesses the impact of citations. Even if the total number of papers or citations may vary, two individuals with similar H-indices could potentially have similar effects on the scientific field. The H-index considers both the number of publications and the number of citations received by those publications, providing a more comprehensive measure of an individual’s research impact (Hirsch, 2005). The M-Index can be used to compare the effects of academics with those with different careers. The number of citations to a document is partly a measure of its scientific impact (Eyre-Walker and Stoletzki, 2013).

CiteSpace is a Java-based tool that visually analyzes academic literature in specific research fields or disciplines. The software allows for analysis and cluster analysis of citations, keywords, authors, institutions, and countries, to visualize scientific research data of specific fields. The key parameters to measure the network structure are betweenness centrality (BC), modularity, and silhouette (Wu et al., 2022). Firstly, the BC metric measures how well one node connects to other nodes on a network path. As a general rule, nodes with BC values greater than 0.1 are key hubs that bridge between nodes, and are also displayed with bright red rings. Secondly, silhouettes can be used to verify that data clusters are consistent. Thirdly, burst detection was used to detect keywords/references that appeared in high numbers for a specific period of time. Moreover, the dual-mapping overlay method in Citespace consists of two parts: elemental mapping and overlay mapping. Overlay mapping uses generated authority literature data, while elemental mapping uses citation distribution maps (Xu and Feng, 2021). Finally, we can analyze trends and emerging priorities in the research field using a network of keywords and co-cited literature datasets.

SCImago Graphica provides a range of graphical tools, such as journal maps and trend graphs that are useful for visualizing research output and trends at the journal and country levels (Hassan-Montero et al., 2014). By revealing the scientific collaboration relationships, strength, and focus among countries using the national collaboration network, this study aims to shed light on the development trends and focal areas of international academic collaboration.

The alluvial generator in MapEquation visualization is used to visualize the flow and correlation relationships between different categories or variables (Rosvall and Bergstrom, 2010; Blöcker et al., 2023). It highlights significant structural changes to reveal the basis and frontiers of network pharmacology research on anti-cancer in literature co-citation network data. The tool helps to connect structural and functional modifications more intuitively.

## Results

### Trend analysis of country-specific annual publication growth

This study includes 3,018 articles, with 2,210 in WoSCC and 808 in PubMed. Among the WoSCC are 2,036 articles and 174 reviews authored by 115,951 authors from 6,319 institutions. These articles were published in 492 journals and cited 69,219 times in 1,014 journals. In addition, there are 748 articles and 60 reviews in PubMed, authored by the same group of authors from the same institutions in the same countries. These articles were published in 492 journals and cited 69,219 times in 1,014 journals.

**FIGURE 2:**
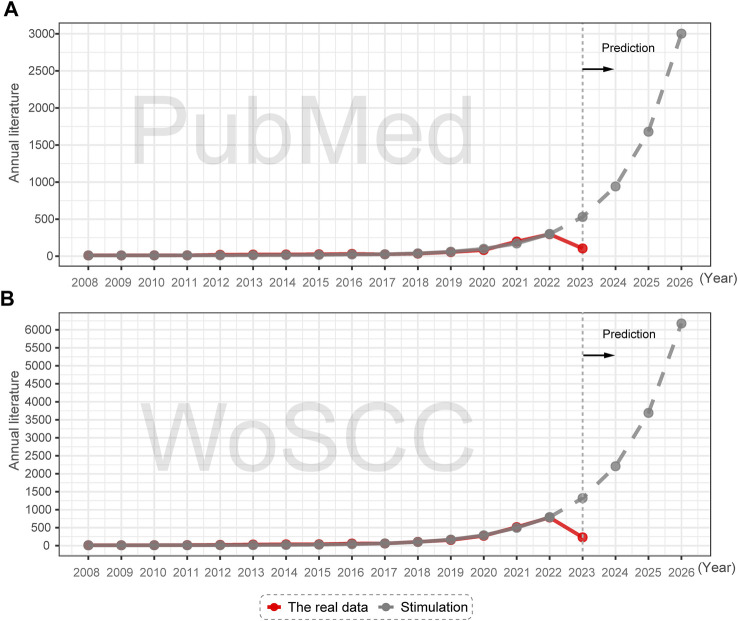
Annual publication trends and future predictions of NPART in PubMed and WoSCC databases. **(A)** GM model indicates significant increases in PubMed publications from 2023 to 2026. **(B)** WoSCC publications from 2023 to 2026 are higher than PubMed publications, but growth trends remain similar.

To a certain extent, the number of publications in a field indicates its developmental scale. As depicted in Figure 2, the yearly research output in network pharmacology and anti-cancer research has maintained a consistent growth rate between 2008 and 2022, indicating a positive developmental trend. Notably, there has been a surge in the volume of publications since 2020, with the number of publications reaching 774 in WoSCC and 287 in PubMed for the year 2022, providing evidence of the growing interest of scholars in this field (Supplementary Table S1).

To gain a comprehensive understanding of the pattern of literature growth, we employed the GM(1,1) model to transform the data sequence into a gray differential equation based on the growth trends of publication output data from 2008 to 2022. We derive the predicted publication output for 2023–2026 by exponentiating the accumulated line. The GM(1,1) model’s accuracy using WoSCC and PubMed data is 0.9953 and 0.9965, respectively, indicating high precision. The Annual scientific publications output in the NPART is consistent with the fitting curve of the model, meaning that the literature output in this field will undergo a rapid growth phase over the next 4 years.

### National and institutional collaborative network analysis

**FIGURE 3:**
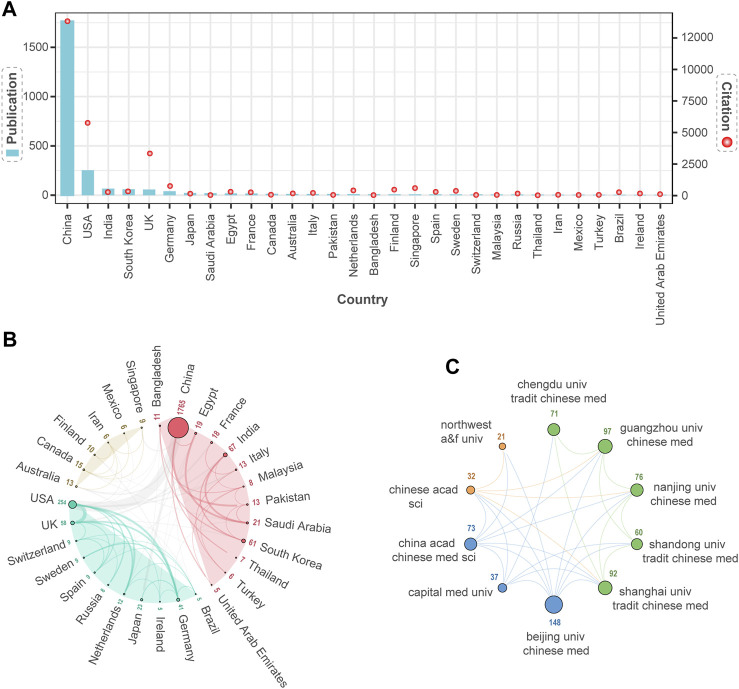
The Distribution of Collaborative Publications and Institutions among Countries. **(A)** Top 30 countries in terms of publication output and citation frequency. **(B)** Three clusters of the national collaborative network are based on similarity of research. China and the United States have the highest level of collaboration intensity. **(C)** Three clusters are formed based on similar research, with extensive collaboration between clusters. Above each node are the institutions’ publications.

To evaluate the performance of a country’s contribution to the NPART field, we first conducted a statistical analysis of the number of articles published by each country. The results showed that China, the United States, and India ranked in the top three. According to Figure 3A, the top three countries in terms of total citation count were China, the United States, and the United Kingdom. Secondly, we used VOSviewer and Scimago Graphica to analyze the collaboration network of the top 30 countries visually. The size of each node was proportional to the number of articles published by that country, with larger nodes indicating a higher number of articles published. Besides, the width of the lines connecting nodes represented the strength of collaboration between countries, with more comprehensive lines indicating closer cooperation. Furthermore, the collaboration network was divided into three clusters, with countries within the same cluster collaborating more closely. Meanwhile, the thickest gray line in different clusters indicated that China and the United States were the leading countries with close collaboration (Figure 3B).

Finally, we can examine the scientific research status of the top 30 countries in the NPART field from the perspective of publications and scientific collaboration. The distribution of publications is uneven among different countries. Regarding the number of publications, China, the United States, Germany, and the United Kingdom are at the forefront of a comprehensive research collaboration network. However, countries like Singapore, Mexico, and Iran are ranked lower.

Supplementary Table S2 provides detailed information on the publications of the top 30 countries, highlighting the variations in collaboration between them. The citation count of a paper reflects its quality. Therefore, China, the United States, and the United Kingdom have conducted in-depth research, resulting in relatively mature outcomes with broad impact. Distributed collaborative research would undoubtedly contribute to the further development of this field if more countries were involved in extensive research.

## Analysis of author contributions and collaborative networks

Analyzing authors in a specific field can evaluate their contributions by identifying core authors. Core authors refer to individuals who have long been engaged in research in the area and have a significant impact on other researchers. According to Price’s law, a specific formula can calculate the minimum number of papers core authors publish. Therefore, identifying core authors is an effective method that can be used to evaluate the contributions of authors in a given field.

$$Price’\;Law\;m=0.749\times \sqrt{n_{\max}}$$

Where m is the minimum number of papers required for a researcher to qualify as a core author. n_max_ represents the maximum number of documents published by the most prolific author. Table 1 lists the top 10 most influential authors contributing to network pharmacology research in anti-cancer studies. The most prolific author in the NPART field is Wu Jiarui from the Beijing University of Chinese Medicine, with 20 published papers. Therefore, the threshold for the number of research articles for core authors is 3.26, meaning that authors who have published four or more pieces are considered core authors. There are 191 core authors, with a total publication count of 1,362 papers, accounting for 61.63% of the total count, reaching half of the standard proposed by Price’s Law.

**TABLE 1:** Top 10 Most effective authors.

Rank	Author	Country	h-index	g-index	M-index	TC	NP
1	Zhang Ying	China	11	27	1.1	827	72
2	Li Yan	China	15	25	1.667	688	64
3	Li Shao	China	14	23	1.273	1,477	23
4	Wang Qi	China	12	21	1.714	476	34
5	Wang Yonghua	China	11	21	1.222	481	29
6	Li Rong	China	11	19	1.375	475	19
7	Wang Yu	China	9	19	0.75	442	69
8	Zhang Bo	China	6	18	0.545	1,033	18
9	Wang Ning	China	12	17	1.714	500	17
10	Popel Aleksander S	United States	12	17	0.857	482	17

**FIGURE 4:**
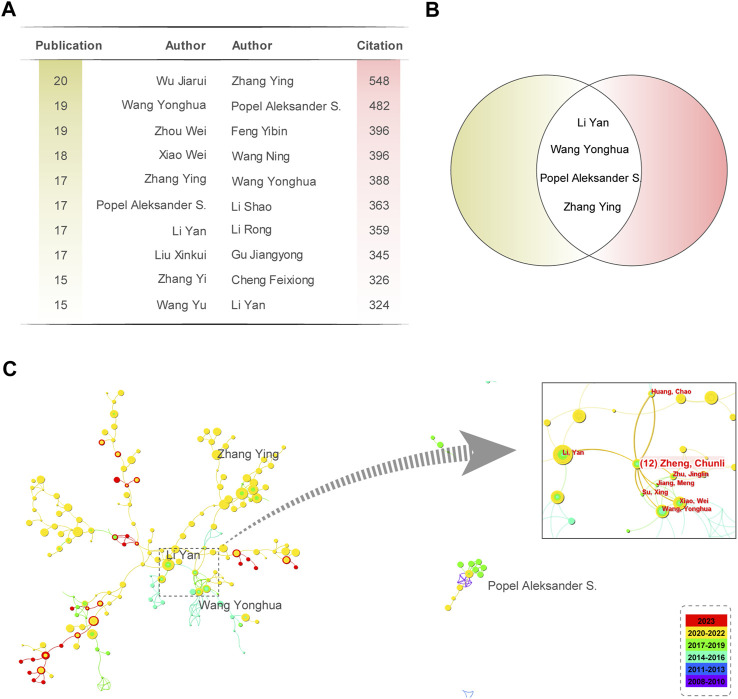
Analysis of the Citations of Authors’ Publications and Collaboration. **(A)** Top 10 authors based on the number of publications and citations. **(B)** Four researchers were identified as the intersection of the Top 10 authors based on the number of publications and citations they have received. **(C)** Collaboration network diagram of 4 authors with high publication and citation counts. Zheng Chunli is an important node for collaboration between Li Yan and Wang Yonghua.

Furthermore, we have identified a relatively stable group of authors in the field of NPART. Supplementary Table S3 provides detailed information about the top 10 highly cited authors. Wang Yonghua and Xiao Wei formed a relatively stable cooperative relationship among highly productive authors. Wu Jiarui published the most papers, with 20 papers and 172 citations, resulting in an average of 8.6 citations per paper. Zhang Ying published 17 papers and obtained 548 citations, with the highest number per article (32.24). Meanwhile, we also compiled the top 10 most productive and cited authors (Figure 4A). By taking the intersection, we identified four scholars, Wang Yonghua, Zhang Ying, Popel Aleksander S., and Li Yan, whose research achievements in network pharmacology for anti-cancer studies are particularly noteworthy (Figure 4B). It is worth noting that Zheng Chunli, as a link between Wang Yonghua and Li Yan, has played an essential role in promoting communication and development in this field (Figure 4C). However, Popel Aleksander S. has a relatively low frequency of collaboration with other groups and needs to strengthen collaboration with them further.

These authors aim to deepen the understanding of the pharmacological mechanisms of TCM in anti-cancer and immune regulation through network pharmacology methods and provide helpful inspiration for developing new anti-cancer drugs from TCM. Their research achievements are expected to provide new treatment strategies and directions for cancer therapy and immune regulation.

### Analysis of keyword Co-occurrence and clustering

Through keyword analysis, we classified closely related keywords in anti-cancer research into distinct groups and linked each with a corresponding theme. In fact, the interconnections between these themes are established through the interconnections between the keywords. This process involved a comprehensive summation of the keywords and the identification of the themes using clustering analysis. Based on this, we can make informed inferences regarding this field’s current research frontiers and trends.

**FIGURE 5:**
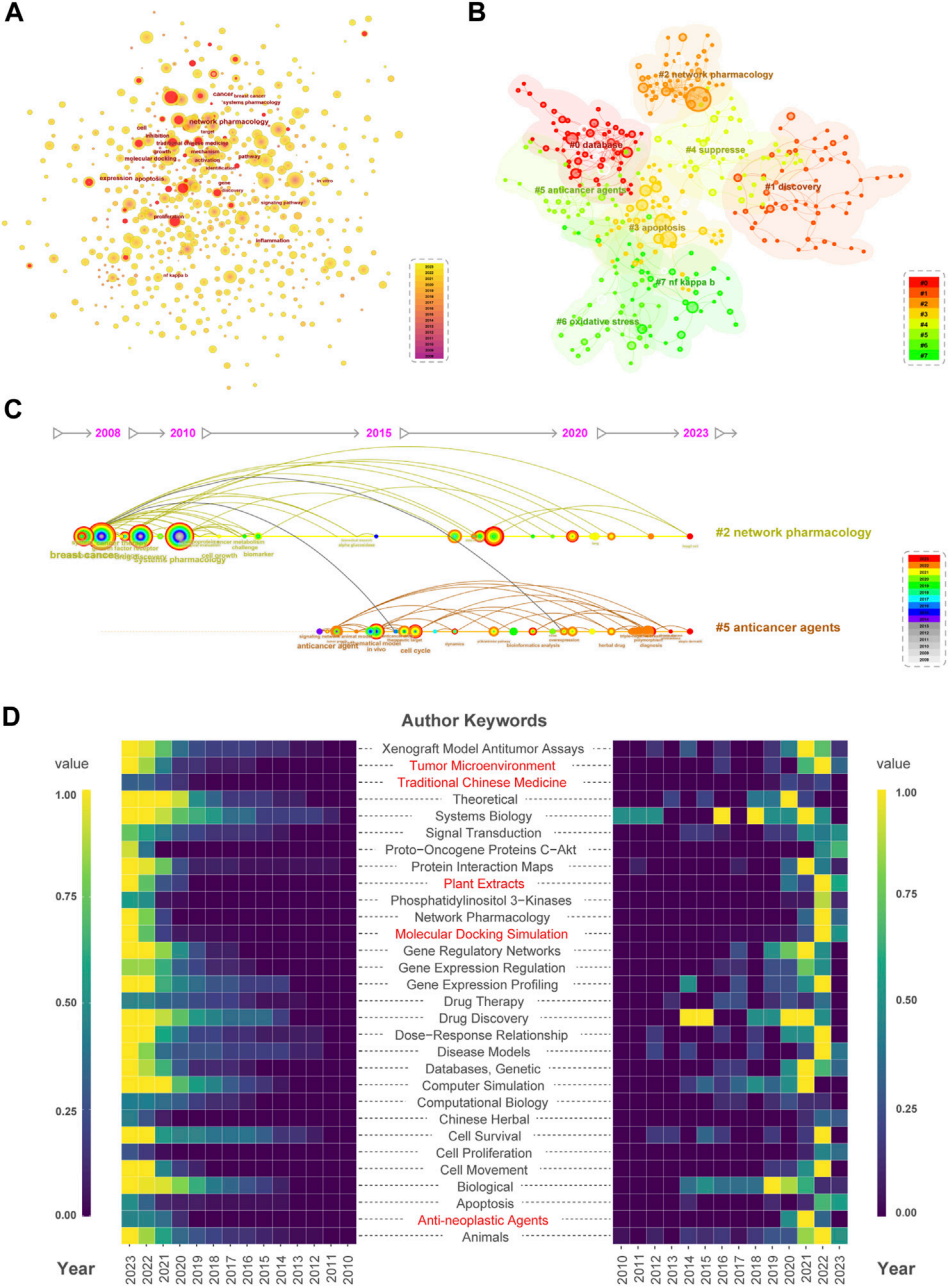
Comprehensive analysis of keywords. **(A)** Keyword co-occurrence and burstness. Node size is directly proportional to keyword frequency. **(B)** Select #0-#7 clusters for keyword clustering analysis. From Cluster #0 to Cluster #7, the number of keywords decreases sequentially. **(C)** Keyword clusters #2 and #5 have a mutual correlation. Cluster #2 exhibited keywords related to breast cancer, growth factor receptor, and cancer therapy in 2008. Cluster #5 featured keywords related to anticancer drugs in 2015. **(D)** Heatmap analysis of the annual and cumulative frequency of keywords in PubMed literature. Red labels indicate emerging research hotspots. Purple to yellow color blocks represent increasing standardized keyword frequency.

This study used Citespace software to generate a keyword co-occurrence visualization map. After combining synonyms and eliminating irrelevant terms, the map contained 679 keywords and 2,793 links. The keywords with a burst phenomenon are represented by central red dots, indicating that the field or theme is rapidly developing or receiving widespread attention (Figure 5A). The appearance of keywords such as “immunotherapy,” “herbal medicine,” “alkaloid,” and “vitamin C″ in the past 3 years suggests the future research direction and hot topics in the NPART field. In addition, we selected the top 20 keywords as core keywords based on their frequency, BC and burst strength (Table 2). Meanwhile, BC is an essential indicator of keyword importance within a network. Keywords with high BC play significant roles in the structure and can be seen as bridges connecting research topics. It is worth noting that “breast cancer” and “mechanism,” as well as “endothelial growth factor” and “angiogenesis,” all have a BC score greater than 0.1. This indicates that these keywords hold prominent positions within the NPART structure. In addition, “tumor necrosis factor” and “anticancer agent” are crucial keywords that cannot be ignored. Their BC scores of 0.09, close to 0.1, also indicate their significant positions within NPART. As a result, identifying these key terms will allow us to better understand which concepts are most relevant to this field.

**TABLE 2:** Top 20 keywords in frequency, betweenness centrality, and strength.

Rank	Keywords	Freq	Keywords	Betweenness centrality	Keywords	Burst	Begin	End
1	network pharmacology	1,064	breast cancer	0.23	systems pharmacology	30.52	2010	2019
2	apoptosis	401	mechanism	0.22	drug discovery	19.98	2009	2018
3	expression	398	discovery	0.18	systems biology	15.67	2012	2019
4	cancer	347	combination therapy	0.18	discovery	15.16	2012	2019
5	molecular docking	307	medicine	0.16	gene expression	12.36	2009	2018
6	cell	300	apoptosis	0.15	mathematical model	9.12	2015	2020
7	traditional chinese medicine	242	drug	0.15	quantitative systems pharmacology	7.61	2017	2020
8	pathway	232	activation	0.14	drug repurposing	6.49	2016	2018
9	inhibition	193	gene expression	0.13	natural product	6.27	2016	2019
10	activation	189	angiogenesis	0.13	network	5.91	2012	2019
11	inflammation	185	biology	0.11	database	5.8	2012	2020
12	mechanism	172	activated protein kinase	0.11	combination therapy	5.54	2008	2019
13	growth	153	endothelial growth factor	0.1	*in vitro*	4.55	2018	2020
14	gene	141	tumor necrosis factor	0.09	resource	4.54	2012	2020
15	systems pharmacology	140	anticancer agent	0.09	prediction	4.5	2019	2020
16	proliferation	136	pathway	0.08	precision medicine	4.41	2017	2018
17	signaling pathway	119	drug development	0.08	drug	4.32	2014	2020
18	*in vitro*	114	cancer	0.07	inhibitor	4.31	2012	2019
19	nf kappa b	113	systems pharmacology	0.07	ursolic acid	4.07	2019	2020
20	breast cancer	110	systems biology	0.07	simulation	3.93	2016	2017

Moreover, co-occurrence frequency is an essential indicator of research hotspots in this field. We can conclude that keywords such as “apoptosis,” “molecular docking,” and “traditional Chinese medicine” still hold important positions in the citation information presented in Table 2. By conducting burst analysis, we can observe that emerging research frontiers often experience explosive growth quickly before either evolving into the following popular topic or disappearing altogether. Thus, by analyzing burst trends over time, we can gain insights into how research topics evolve within this field and identify potential areas for future investigation. Over the past 5 years, NPART has exhibited a trend towards diversification and has attracted broad attention in several fields such as “*invitro*”, “prediction”, “database”, “combination therapy”, and “natural product.” At the same time, keywords like “drug discovery,” “systems pharmacology,” and “systems biology” continue to be areas of future research interest. These findings suggest that the field of NPART is continually evolving and expanding into new frontiers. As researchers continue to explore these emerging topics while also investigating established ones, we can expect future advancements in this field.

The visualization of keyword clustering in Figure 5B provides a clear overview of the topics studied in this field and their interrelationships. At the same time, we use a timeline to represent the occurrence of each keyword. This is particularly helpful in understanding the evolution of these themes. Furthermore, the silhouette and module scores confirm the validity of the clustering results. The connection between “combination therapy” (Nelander et al., 2008) in the #2 topic and “anticancer drug” (Kong et al., 2016) in the #5 topic suggests that these two concepts are related, as they both appeared during a specific period (Figure 5C). This finding may be helpful for researchers who are interested in developing new anticancer therapies. Additionally, their temporal continuity makes it possible to distinguish classical topics from emerging ones. Standard topics such as “#0 database”, “#1 discovery”, “#2 network pharmacology”, “#3 apoptosis”, and “#4 suppress” show good temporal continuity, meaning they have been studied for an extended period with sustained interest from scholars. In contrast, emerging topics like “#5 anticancer agents”, “#6 oxidative stress”, and “#7 nf kappa b” have recently attracted attention but require more research to establish themselves within the field.

In addition, we can identify current research hotspots such as “Tumor Microenvironment”, “Traditional Chinese Medicine”, “Plant Extracts”, “Molecular Docking Simulation”, and “Anti-neoplastic Agents” using heatmaps to visualize frequency accumulation across all documents for each keyword (left panel), as well as occurrence counts for different years (right panel). We conducted an annual keyword analysis of relevant literature in the PubMed database to predict future research trends. This serves both as a supplement and a validation to the keyword hotspot analysis results from WoSCC database. As a result, the above keywords could become hotspots and trends for future research in this field.

## Analysis of journals and co-cited journals

We utilized the Bibloshiny platform to identify high-impact journals in the NPART field and conducted a visual analysis using CiteSpace. Table 3 displays the top ten journals ranked by their exceptional performance in terms of publications and co-citations. These journals are distributed across the JCR’s Q1, Q2, Q3, and Q4 categories, with three having an impact factor higher than 5. Furthermore, the table includes their corresponding impact factors (JCR, 2021) and JCR categories. Notably, Frontiers in Pharmacology and Evidence-Based Complementary and Alternative Medicine have publication numbers exceeding 200, indicating their prominence and activity in the field compared to other journals. The journal with the highest number of publications is “Frontiers in Pharmacology” (impact factor of 5.998, JCR category of Q1), with a total of 205 articles. Close behind is “Evidence-Based Complementary and Alternative Medicine,” with an impact factor of 2.65 and a JCR category of Q3. It has a total of 204 articles. Additionally, we have the “Journal of Ethnopharmacology” with an impact factor of 5.195 and a JCR category of Q2, featuring 120 articles.

**TABLE 3:** The number of publications, IF (JCR 2021), and JCR quartile of the top 10 and co-cited journals.

Rank	Journal	Publication	IF(JCR 2021)	JCR quatile	Co-cited journal	Citations	IF(JCR 2021)	JCR quatile
1	FRONTIERS IN PHARMACOLOGY	205	5.988	Q1	BIOMEDICINE and PHARMACOTHERAPY	287	7.419	Q1
2	EVIDENCE-BASED COMPLEMENTARY AND ALTERNATIVE MEDICINE	204	2.65	Q3	BIOMED RESEARCH INTERNATIONAL	133	3.246	Q3
3	JOURNAL OF ETHNOPHARMACOLOGY	120	5.195	Q2	JOURNAL OF PHARMACEUTICAL AND BIOMEDICAL ANALYSIS	74	3.571	Q2
4	SCIENTIFIC REPORTS	55	4.997	Q2	ANNALS OF BIOMEDICAL ENGINEERING	36	4.219	Q2
5	NATURAL PRODUCT COMMUNICATIONS	52	1.496	Q4	ANNUAL REVIEW OF BIOMEDICAL ENGINEERING	22	11.324	Q1
6	MEDICINE	45	1.817	Q3	BIOMEDICAL REPORTS	10	0.48	Q3
7	PHYTOMEDICINE	44	6.656	Q1	BIOMEDICINES	8	4.757	Q2
8	BIOMED RESEARCH INTERNATIONAL	36	3.246	Q3	BIOMEDICAL CHROMATOGRAPHY	7	1.911	Q4
9	DRUG DESIGN DEVELOPMENT AND THERAPY	35	4.319	Q2	ASIAN PACIFIC JOURNAL OF TROPICAL BIOMEDICINE	6	1.514	Q3
10	BMC COMPLEMENTARY MEDICINE AND THERAPIES	34	2.838	Q1	COMPUTER METHODS AND PROGRAMS IN BIOMEDICINE	4	7.027	Q1

In addition, three journals commonly cited by the top 10 journals have an impact factor exceeding 5. “Annual Review of Biomedical Engineering” ranks first with an impact factor of 11.324 and 22 citations, followed by “Biomedicine and Pharmacotherapy” (impact factor of 7.419, 287 citations) and “Computer Methods and Programs in Biomedicine” (impact factor of 7.027, 4 sources).

**FIGURE 6:**
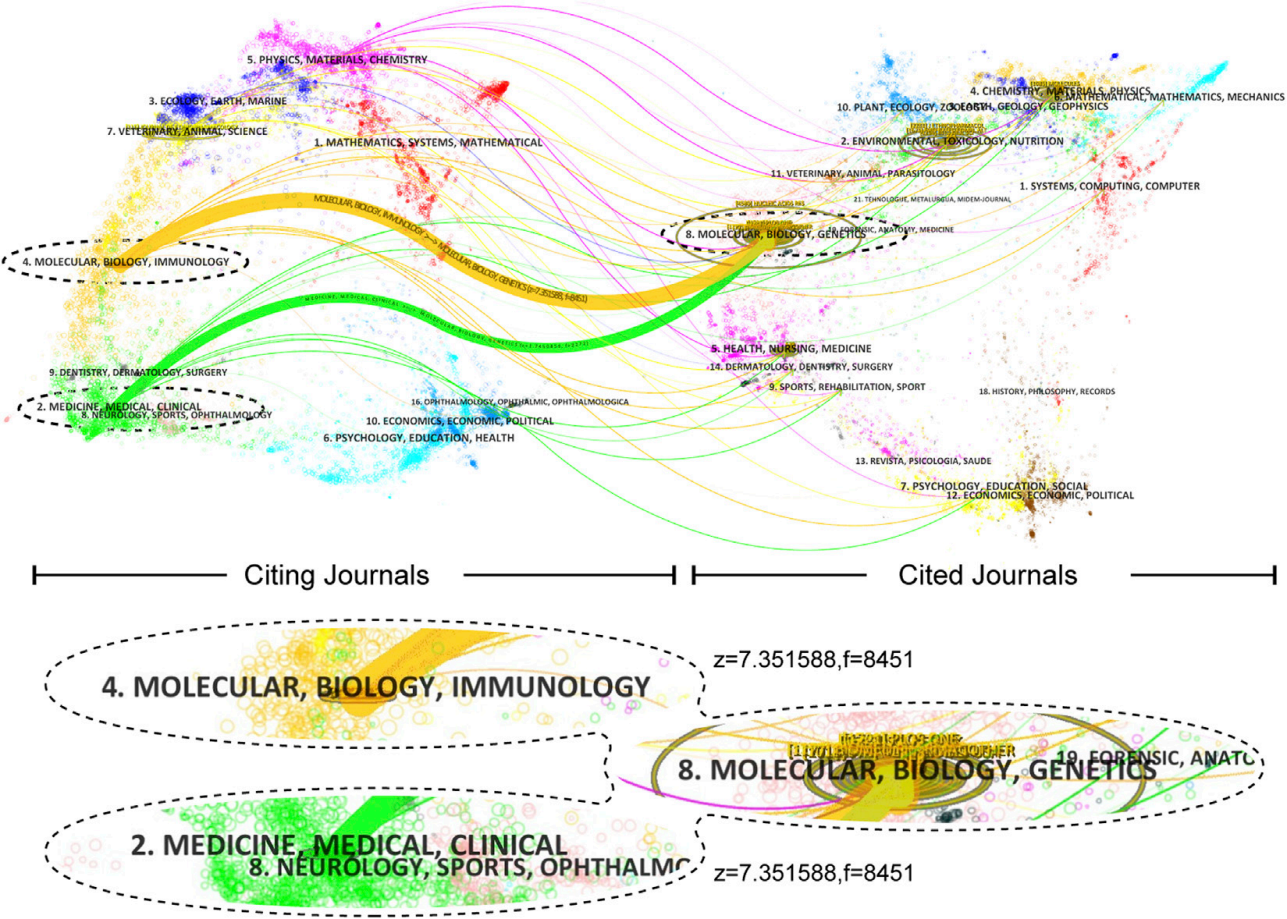
A dual-map overlay shows journals. Z-scores standardize citation frequency for comparative citation analysis by mitigating disciplinary differences. Citation paths are displayed by discipline, with widths proportional to z-score-scaled citation frequency. Labels represent research subjects, while the wavy curve connects citing articles (on the left) and cited articles (on the right).

The cited journals play a crucial role in providing the necessary knowledge base for the citing journals. The yellow path in the citation network indicates that research published in journals related to “Molecular Biology and Immunology” tends to cite journals in the main field of “Molecular Biology and Genetics.” Similarly, the light green path suggests that research published in journals related to “Medicine, Healthcare, and Clinical” also tends to cite journals in the main field of “Molecular Biology and Genetics.” These findings highlight the interconnectedness of different areas and the importance of cross-disciplinary research in advancing scientific knowledge (Figure 6).

### Literature co-citation analysis identifies hotspots and frontiers

For literature to be cited together, it must possess similar content. When two or more studies subsequently cite the same articles, a co-citation relationship is established between them. Co-citation reference analysis is a method for measuring the relationship between reference literature. Therefore, this method can uncover research themes and evolutionary trends within a particular field by incorporating cluster analysis.

**FIGURE 7:**
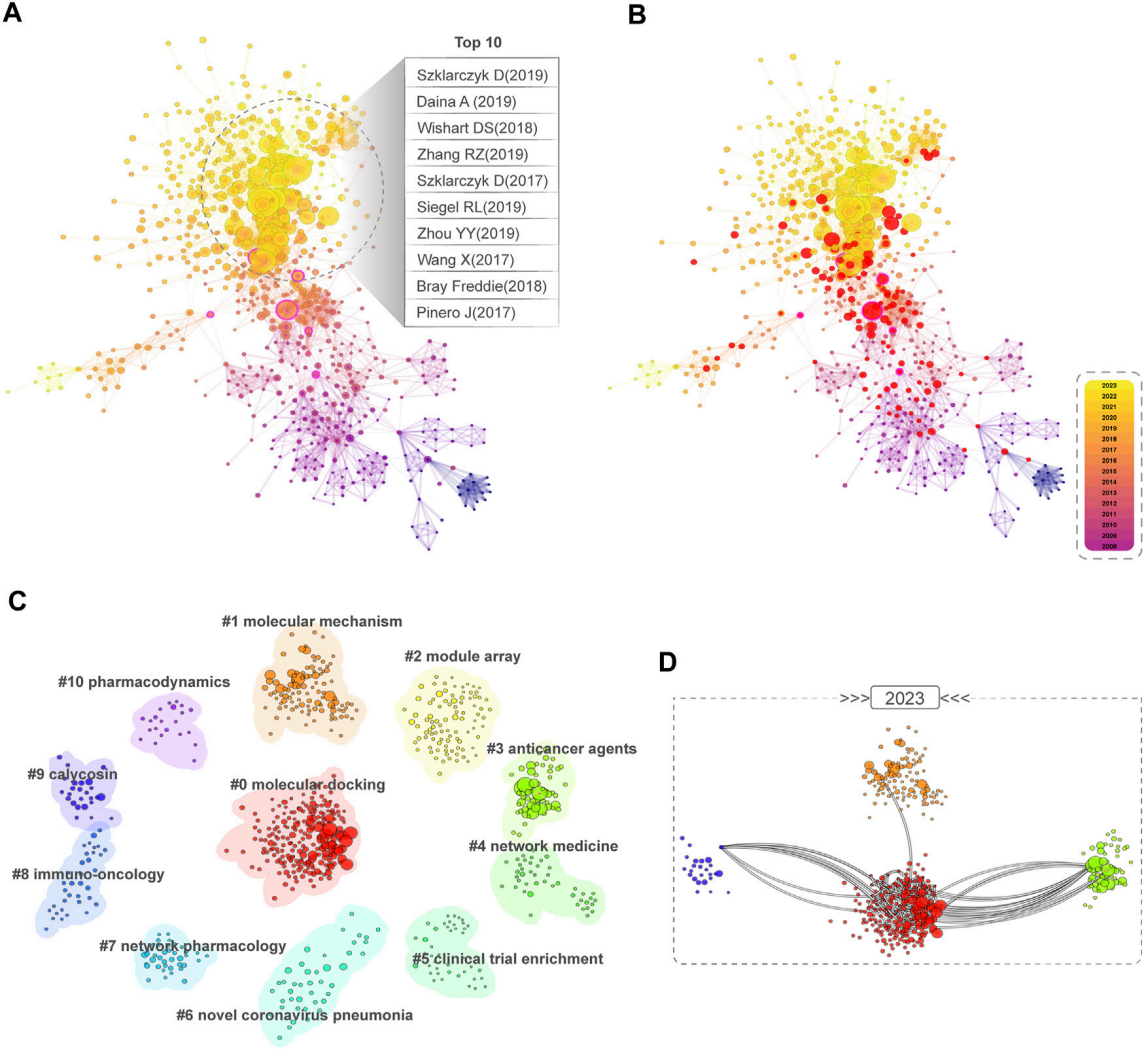
Co-citation and clustering analysis network. **(A)** Co-citation reference network among BC. The size of the article node is proportional to the number of its co-citations, while nodes with high BC are marked by a purple outer ring. **(B)** Co-citation reference network with a burst of citations. Burst nodes are labeled with a red circle. **(C)** A circular view of the cluster of #0-#10 literature commonly cited, obtained through reference clustering based on the similarity between references. **(D)** Clusters #0, #1, #4, and #9 show a dense connection in 2023, indicating cutting-edge research directions.

Figure 7A displays the top 10 most cited articles and utilizes CiteSpace to present their co-citation network. In Figure 7A, nodes with high BC are marked in purple outer rings, while in Figure 7B, nodes with citation bursts are marked in red circles. Additionally, we identified the top 25 articles related to NPART with the most robust citation bursts shown in Supplementary Figure S2. These articles were classified into 11 categories (Supplementary Table S4), and their degree of correlation served as the basis for cluster classification (Figure 7C). The most significant cluster was “#0 molecular docking”. In contrast, the earliest research cluster was “#5 clinical trial enrichment”. In 2023, it was discovered that there was a high frequency of connections among different groups, such as “#0 molecular docking”, “#9 calycosin”, “#1 molecular mechanism”, and “#3 anticancer agents” (Figure 7D).

**FIGURE 8:**
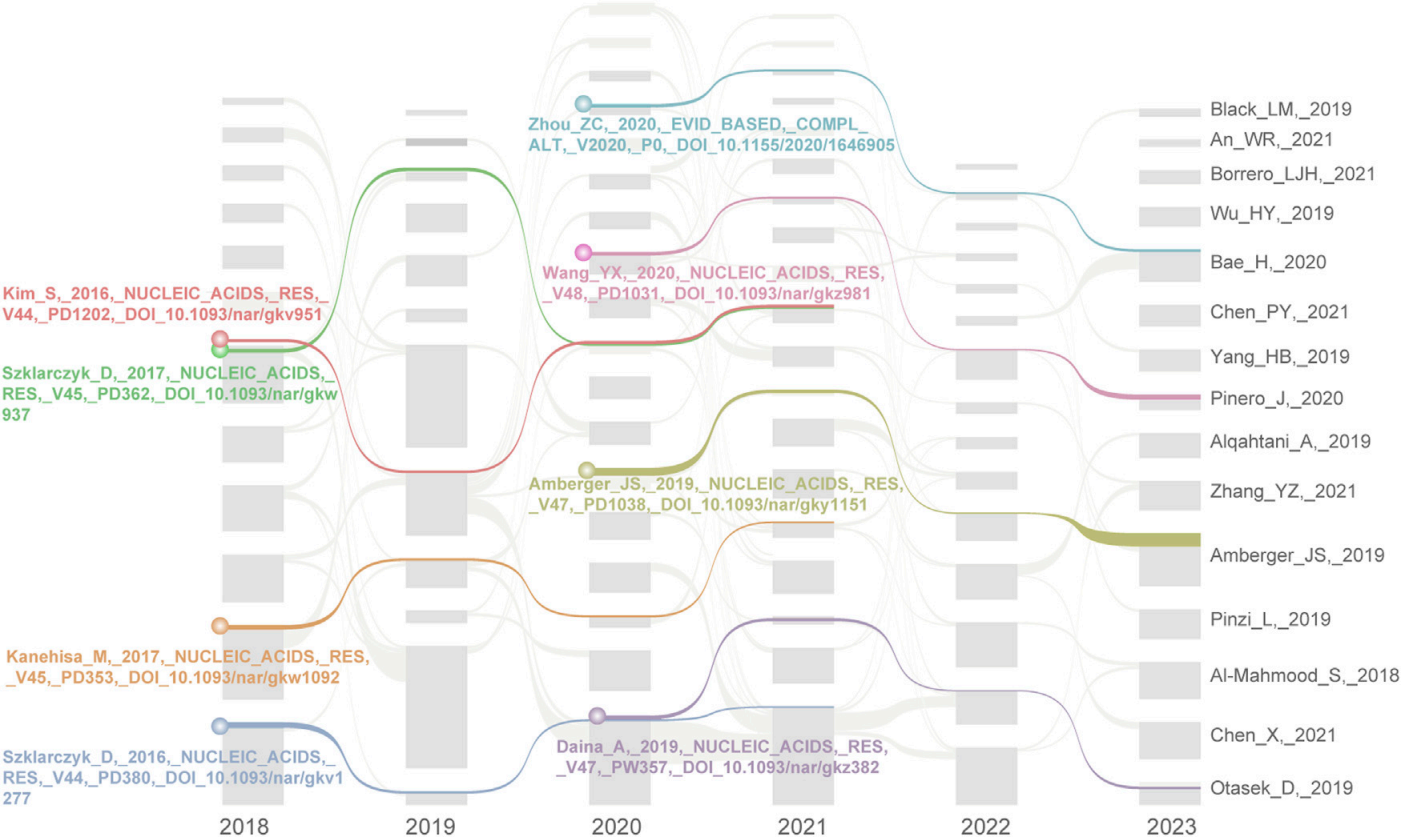
The alluvial flow map of landmark literature between 2018 and 2023. During their individual citation impact periods of 4 years, these 8 papers made significant contributions to NPART research. In particular, Szklarczyk_D and Daina_A papers demonstrate strong continuity and lasting influence.

In addition, we generated a literature citation network using Citespace from 2018 to May 2023 to explore the key literature on NPART. Subsequently, we imported the network information files for each year into the streamflow generator program and obtained a streamflow map of landmark literature following filtering and layout adjustments. Figure 8 shows four highly acclaimed milestone articles with a streamflow continuity of 4 years between 2018–2021 and 2020–2023.

Between 2018–2021, four papers were published on the topic, with the first being Kim et al., 2016 publication “PubChem Substance; Compound databases” (Kim et al., 2016). This literature delves into PubChem as a public chemical substance and biological activity information database, offering chemical information resources and search interfaces, supporting data submission and programming access, and featuring derivative resources such as PubChem3D and PubChemRDF. In network pharmacology, these resources are utilized for drug discovery and design. Secondly, Kanehisa_M introduced KEGG as an encyclopedia of genes and genomes, encompassing the KO database, networks of molecular interactions, reactions and relations, BRITE hierarchy, and KEGG modules (Kanehisa et al., 2017). In fact, KEGG has proven to be a valuable tool for predicting drug action mechanisms, identifying new drug targets, and designing novel drug molecules. Through enhancements to the DISEASE and DRUG databases, KEGG can integrate diseases and drugs with molecular networks. Thirdly, Szklarczyk_D developed the STRING and STITCH5 databases for NPART development. STRING is a valuable tool for predicting drug action mechanisms and identifying new targets (Szklarczyk et al., 2017). By analyzing the interactions between drugs and proteins, it is possible to predict the mode of action of drugs and potential side effects. This makes STRING an essential resource for drug discovery and development. Furthermore, the STITCH5 database is also a valuable resource that integrates multiple data sources, including interaction information between proteins and small molecules, covering 430,000 chemical substances (Szklarczyk et al., 2016). It provides a new network view that allows users to view the binding affinity of chemical substances in the interaction network, thereby facilitating a quick understanding of the potential impact of chemical substances on their interaction partners. This makes STITCH5 a valuable tool for drug discovery and design. In summary, these tools discussed above are of immense significance for network pharmacology research, particularly in anti-cancer research. These articles represent classic works of original network pharmacology research and serve as the foundation of this field.

In addition, the methods and tools proposed in the four critical articles from 2020 to 2023 have gained widespread attention and recognition for their frontier nature and innovativeness in the NPART. These articles represent important research achievements in this domain, which are significant for researchers and clinicians seeking insights into the network pharmacological mechanisms underlying anti-cancer treatment strategies. Firstly, Zhou_ZC has pointed out that network pharmacology can be employed to elucidate the multi-target characteristics of traditional Chinese medicine, particularly for treating complex diseases (Zhou et al., 2020). Furthermore, network pharmacology offers a novel research approach to investigating traditional Chinese medicine. Secondly, Wang_YX established the Therapeutic Target Database, which encompasses the regulatory effects of microRNAs and transcription factors on targets, protein-target interactions, patented drugs, and their corresponding targets (Wang et al., 2019). These data are derived from other validated, clinically tested, literature-reported target collections. Thirdly, OMIM.org is a website constructed by Amberger_JS to integrate the knowledge of phenotype-gene relationships. The website documents have known genetic diseases and are a primary repository for information on gene-phenotype associations (Amberger et al., 2019). Lastly, the Swiss Target Prediction tool aims to predict protein targets of small molecules (Daina et al., 2019). The device has a collection of 376,342 compounds with known experimental activity and searches for similar 2D and 3D molecules to predict 3,068 large molecule targets. The latest version has a predictive performance of over 70% and can correctly predict at least one human prey in the top 15 predictions. The tool has been applied in anti-cancer research to predict the interactions between small molecules and tumor-related proteins and guide drug design.

In summary, references with high BC, high citation counts, and citation durations of 4 years are essential to advance our understanding of NPART. In addition, the evolutionary relationship between the cited literature clusters suggests that “#0 molecular docking”, “#1 molecular mechanism”, “#4 network medicine”, and “#9 calycosin” may become hotspots and frontiers.

## Discussion

This study employed various bibliometric tools to analyze the publications on NPART from the WoSCC and PubMed databases from 2008 to May 2023. This is the first paper that applies bibliometric analysis to network pharmacology in anti-cancer research. We summarized the annual publication output, countries, institutional affiliations, and collaborative networks of authors involved in NPART. Additionally, utilizing citespace6.2. R4 and the BiblioMetrix R package, we conducted keyword frequency analysis and co-occurrence analysis to uncover the research hotspots and trends in this field. Furthermore, we performed cluster analysis on the co-citation relationships among the publications to reveal the main research directions and academic focal points within this domain. Lastly, we examined the significant application value of network pharmacology in anti-cancer research and discussed its challenges.

## Summary of main findings

By May 2023, there has been an overall increasing trend in the annual publication output and citation frequency of NPART-related research, reflecting the sustained interest and diversity within the field. In addition, China and the United States emerged as the main contributors in this domain. China has the highest number of publications, while the United States demonstrates the highest citation frequency and the most extensive collaborations with other countries or regions. Figure 3C illustrates that among the top ten institutions in publication output, Beijing University of Chinese Medicine, Guangzhou University of Chinese Medicine, and Shanghai University of Traditional Chinese Medicine have contributed to more than 90 articles. Beijing University of Chinese Medicine is the most productive institution within the collaborative network, exhibiting the highest overall link strength. These achievements closely correlate with the support provided by Chinese institutions in terms of policies and funding, fostering in-depth research in this field. Moreover, the institutions with the highest betweenness BC are Johns Hopkins University, Nanjing Medical University, and Beijing University of Chinese Medicine, which significantly influence network pharmacology in anti-cancer drug research. Johns Hopkins University first appeared in the institutional network 2010 as a crucial mediator for information flow and knowledge exchange.

Regarding publication output, Frontiers in Pharmacology emerges as the top-ranked journal, while Biomedicine and Pharmacotherapy stands out as the most highly cited journal based on co-citations. It is evident that considering the number of published articles and co-citation counts, the top ten journals focus on different research areas, indicating the broad utilization of NPART across various domains of investigation. Notably, the study by Szklarczyk D titled “STRING v11: protein-protein association networks with increased coverage, supporting functional discovery in genome-wide experimental datasets,” updated in 2019 and published in Nucleic Acids Research. This paper received the highest number of co-citations, signifying the significant attention of scholars in this field towards this particular study (Szklarczyk et al., 2019). Furthermore, Li Shao is a highly cited author with an H-index of 14 (Table 1). Meanwhile, Wu Jiarui ranks first in terms of the number of published articles. Wu Jiarui’s significant contribution to NPART can be seen in the first co-authorship citations made with other collaborators. In recent years, Zhang Ying et al. have emerged as key authors in this field and garnered substantial citation rates. First, they isolated hydroxystaurosporine (OPB) from the Huanglian. Next, they screened potential targets, pathways, and genes for non-small cell lung cancer (NSCLC) through network pharmacology. Finally, the key target for the treatment of NSCLC was validated by molecular docking of OPB to the core target for the treatment of NSCLC (Zhang et al., 2021). Recently, Liu Yang et al. discovered that quercetin from Astragalus membranaceus inhibits the proliferation and migration of ovarian cancer cells by targeting tumor proteins p53, MYC, vascular endothelial growth factor, phosphatase and tensin homolog, AKT serine/threonine kinase, and Cyclin D1. Meanwhile, combining quercetin and olaparib synergistically enhances the anticancer effects against ovarian cancer (Liu et al., 2023). According to reports, tanshinone IIA, a compound derived from Salvia miltiorrhiza, exerts inhibitory effects on the malignant proliferation of prostate cancer by suppressing multiple pathways (Li et al., 2022). It is evident that the field is experiencing rapid and dynamic development as a result of the emergence of new researchers and their significant findings.

## Current research landscape and prevailing issues

Following the bibliometric analysis of the retrieved documents, we discuss the research status and existing problems in network pharmacology research for anticancer studies. The current research primarily focuses on exploring the mechanisms of action between drugs and cancer network targets from the perspective of traditional Chinese medicine based on network pharmacology. Our discussion will focus on two aspects.

On the one hand, the research methodology in anticancer studies using network pharmacology has evolved from initially relying solely on a single database to gradually incorporating cross-validation using multiple databases. Data is combined from multiple databases in this paradigm shift in research integration to ensure quality and reliability. In addition, it provides multidimensional analyses that reveal crucial drug targets in cancer and facilitate drug development. As a result of this transformation, five notable advantages have emerged:

1. Collecting data from different database sources can provide more comprehensive and diverse information, allowing for a broader range of pharmacological information covering more compounds, targets, and bioactivity data.
2. Cross-validating data from multiple databases eliminates potential errors and biases, and improves the credibility of study results.
3. Using multiple database sources improves accuracy and reliability because low-quality or inconsistent data can be filtered out.
4. The information provided by different databases can cover a wide range of topics, such as gene expression, protein interactions, and metabolic pathways.
5. A multi-database integration process facilitates the discovery of potential drug targets. In turn, this leads to the discovery of new therapeutic targets and drug designs.

Despite this, network pharmacology research on anticancer faces some problems. These are we summarized below:

1. Integration is challenging due to the significant and independent differences in data volumes between TCM databases.
2. The algorithms for analyzing network models are diverse and lack uniformity.
3. Animal and cellular validations are lacking in some studies.

On the other hand, Chinese medicines have received academic attention for their advantages over synthetic drugs, such as low toxicity, multi-targeting, and lower environmental impact. Ginsenosides in Panax ginseng suppress multiple pivotal enzymes and proteins associated with ovarian cancer (Sun and Singh, 2023). Therefore, these substances may be prospective therapeutic targets. In addition, CKI is widely used in digestive tract tumors, such as pancreatic and esophageal cancer (Guo et al., 2023). Meanwhile, Wu et al. found that the molecular mechanism of CKI treatment for pancreatic cancer is closely related to five core genes associated with essential signaling pathways and survival-related genes (Wu et al., 2021). Furthermore, Scopoletin is a natural compound belonging to the coumarin class. It inhibits the RAS-RAF-MEK-ERK pathway and the PI3K/AKT pathway by inhibiting the activity of epidermal growth factor receptor, thereby inducing apoptosis and inhibiting the proliferation of non-small cell lung cancer cells. In summary, TCM anticancer mechanisms are moving from database-based computational simulation to experimental validation in combination with cell biology in network pharmacology research. It suggests that research methodology is evolving in this field.

### Future hotspots and research trends

We also analyzed the overall evolutionary trends in this field. From keywords perspective, network pharmacology and cancer have emerged as core and foundational research components in the entire domain. Notably, keywords such as, traditional Chinese medicine, and anti-cancer research have gained prominence in recent years, indicating the significant research potential in these areas and encouraging scholars to explore them further. Regarding the clustering of keywords in the articles, clusters “#0 databases” and “#5 anticancer agents,” were among the earlier and larger keyword clusters (Figure 5B). The emerging keywords in this field include traditional Chinese medicine, tumor microenvironment, and Phosphatidylinositol 3-Kinases (Figure 5D). In this perspective, scholars have been studying the connection between network pharmacology and anti-cancer and its microenvironment since 2016, and the popularity of these keywords has persisted. Recent studies have demonstrated that ginger regulates the proliferation, migration, and apoptosis of triple-negative breast cancer cells by modulating signaling pathways such as TNF, IL-17, FOXO, MAPK, PI3K/AKT, and others (Luo et al., 2023). In addition, tumor radiotherapy often leads to the development of radiation-induced oral mucositis in patients. A formulation consisting of extracts from *Lonicera japonica*, Paeonia lactiflora, and Cornus officinalis has been employed for the treatment of radiation-induced oral mucositis, targeting multiple pathways, including Toll-like receptor signaling and TNF signaling cascades (Sheng et al., 2023). In summary, Chinese herbal medicine, as an integral part of traditional medicine, demonstrates promising anti-cancer activity and therapeutic potential in cancer treatment. Network pharmacology research enables us to understand Chinese herbal medicine’s active constituents, targets, and mechanisms of action, thereby providing a scientific basis for its application in cancer therapy.

By analyzing the literature citation count, we can identify research hotspots and cutting-edge fields that have garnered widespread attention and citation in the academic community (Wang J. et al., 2023). The 11 clusters identified from the literature citations cover areas such as traditional Chinese medicine, tumor immunology, and clinical trials, indicating that this field has attracted extensive attention from interdisciplinary scholars. The academic influence of these clusters extends beyond individual disciplines, fostering interdisciplinary collaborations and knowledge exchange and driving cross-disciplinary integration and innovation (Tang et al., 2023). The compound Chinese medicine Xihuangwan exerts inhibitory effects on the progression of breast cancer by targeting multiple proteins involved in the inflammatory microenvironment signaling pathways, including FOS, MYC, JUN, PPARG, MMP9, PTGS2, and SERPINE1 (Wu et al., 2020). By applying a network pharmacology approach, Guo et al. identified five components of CKI that may target 187 esophageal cancer-related genes and inhibit genes in cancer cell-associated pathways. The active ingredients identified in the compound injection include quercetin, flavonoids, nobiletin, lignans, and β-sitosterol. These active ingredients have shown potential in treating esophageal cancer (Guo et al., 2023).

In particular, the cluster analysis in 2023 indicates a close relationship and interdependence between cluster #0 (molecular docking), cluster#1 (molecular mechanism), cluster #3 (anticancer agents), and cluster #9 (calycosin), reflecting the current research trends and development directions of NPART. Calycosin, a primary active ingredient isolated from Astragalus membranaceous (Huangqi), has been shown to exhibit significant therapeutic effects on colon cancer by inhibiting the ERK1/2 signaling pathway (Hu et al., 2023). It is worth noting that two distinguished traditional Chinese medicine physicians have invented a core formula comprising Astragalus membranaceous (Huangqi), Salvia miltiorrhiza (Danshen), Carthamus tinctorius seeds (Suhonghuazi), Hedyotis diffusa (Baihuasheshecao), Scutellaria barbata (Banzhilian), and Curcuma zedoaria (Ezhu). This formula may exert anticancer effects by targeting 14 liver cancer-related genes, primarily inhibiting inflammation and angiogenesis and enhancing the immune response (Bochuan et al., 2023).

In summary, we applied keyword and literature co-citation cluster analysis to reveal that the current research hotspots mainly focus on databases, herbal medicines, molecular docking, and tumor immune microenvironment regulation. Furthermore, the latest publications indicate that Chinese herbal medicines are developing into compelling cutting-edge research hotspots for fundamental research and clinical application. These research directions provide new perspectives and opportunities for the further development of Chinese herbal medicine anti-tumor therapy.

### Application value and challenges

Although network pharmacology holds significant value in cancer treatment, it still faces challenges regarding data quality, algorithm selection, and reproducibility. From the perspective of application value, network pharmacology demonstrates tremendous potential in anticancer therapy. First of all, network pharmacology can identify key molecular targets and pathways involved in tumor development and progression by integrating various data sources such as genomics, proteomics, and metabolomics. Secondly, it can also predict the efficacy and toxicity of candidate drugs and optimize drug combinations for personalized treatment. Lastly, network pharmacology provides a systems-level understanding of drug interactions with targets, which is crucial for developing safer and more effective anticancer therapies.

However, some challenges need to be addressed when applying network pharmacology to anticancer treatment:

1. The integration and analysis of large-scale omics data require advanced computational and statistical methods, which can pose technical difficulties for researchers.
2. The lack of comprehensive and accurate databases on drug-target interactions and network topology may limit the accuracy and reliability of network pharmacology predictions.
3. The heterogeneity and complexity of tumors can lead to different responses to the same treatment, necessitating the development of personalized and precision anticancer therapies.

In conclusion, network pharmacology holds tremendous potential in anticancer treatment, but some challenges must be addressed. Future research should focus on developing more advanced computational and experimental methods, improving data quality and availability, and exploring personalized and precision medicine strategies.

### Limitations

There are several limitations to this study despite these interesting findings. First, the data for this study were obtained from WoSCC and PubMed databases analyzed separately. Additionally, the Scopus database was not used in this study due to its restricted availability. Secondly, the WoSCC literature contains comprehensive information and is often considered the most appropriate bibliometric database (Cheng et al., 2022a; Chen et al., 2022; Zhong and Lin, 2022). However, the PubMed database lacked information on cited literature and could not be analyzed for literature co-citation. Finally, due to the limitations of these bibliometric software, the specific parameter settings for the relevant analyses are somewhat subjective. This means that they may miss potentially high-impact studies.

## Conclusion

This study can help researchers identify the trends and frontier hotspots in NPART from 2008 to May 2023. NPART is gaining increasing attention from scholars worldwide, and the number of related publications is significantly increasing. Several vital areas show continuous development and progress among the current research hotspots. Firstly, the continuous updating of databases provides rich information on compounds, targets, and diseases for network pharmacology research on anti-cancer. Secondly, the development of traditional Chinese herbal medicine has become an important direction in NPART. Studies focus on the active ingredients, targets, and mechanisms of action of Chinese herbal medicine and their potential in cancer treatment. Meanwhile, “Plant Extracts”, “Calycosin”, and “Molecular Docking Simulation” may become potential hotspots. Finally, the study of the tumor microenvironment has received significant attention. In conclusion, researchers can gain a better understanding of the major countries, organizations, contributors, and journals that are associated with NPART. In addition, it provides researchers with information about research hotspots and trends in the field.

## Data Availability

The original contributions presented in the study are included in the article/Supplementary Material, further inquiries can be directed to the corresponding authors.

## Abbreviations

NPART, network pharmacology research in anti-cancer; WoSCC, Web of Science Core Collection; TCM, traditional Chinese medicine; BC, betweenness BC; CKI, Compound kushen injection; MeSH, Medical Subject Headings; GM, grey prediction model; OPB, hydroxystaurosporine; NSLCC, non-small cell lung cancer.

## References

[B1] Amberger J. S. Bocchini C. A. Scott A. F. Hamosh A. (2019). OMIM.org: Leveraging knowledge across phenotype–gene relationships. Nucleic Acids Res. 47, D1038-D1043–D1043. 10.1093/nar/gky1151 30445645 PMC6323937

[B2] Aria M. Cuccurullo C. (2017). bibliometrix: An R-tool for comprehensive science mapping analysis. J. Informetr. 11, 959–975. 10.1016/j.joi.2017.08.007

[B3] Blöcker C. Smiljanić J. Scholtes I. Rosvall M. (2023). Similarity-based link prediction from modular compression of network flows. 10.48550/arXiv.2208.14220

[B4] Bochuan W. Yong Z. Qiuyun Z. Zhiqiang Z. Changyong L. Zhendong W. (2023). Reveal the mechanisms of prescriptions for liver cancer’ treatment based on two illustrious senior TCM physicians. J. Tradit. Chin. Med. 43, 188–197. 10.19852/j.cnki.jtcm.20221013.001 36640012 PMC9924736

[B5] Chen B.-S. Wu C.-C. (2013). Systems biology as an integrated platform for bioinformatics, systems synthetic biology, and systems metabolic engineering. Cells 2, 635–688. 10.3390/cells2040635 24709875 PMC3972654

[B7] Chen Y. Lin M. Zhuang D. (2022). Wastewater treatment and emerging contaminants: Bibliometric analysis. Chemosphere 297, 133932. 10.1016/j.chemosphere.2022.133932 35149018

[B8] Cheng K. Guo Q. Shen Z. Yang W. Wang Y. Sun Z. (2022a). Bibliometric analysis of global research on cancer photodynamic therapy: Focus on nano-related research. Front. Pharmacol. 13, 927219. 10.3389/fphar.2022.927219 35784740 PMC9243586

[B9] Cheng K. Zhang H. Guo Q. Zhai P. Zhou Y. Yang W. (2022b). Emerging trends and research foci of oncolytic virotherapy for central nervous system tumors: A bibliometric study. Front. Immunol. 13, 975695. 10.3389/fimmu.2022.975695 36148235 PMC9486718

[B10] Cooper I. D. (2015). Bibliometrics basics. J. Med. Libr. Assoc. 103, 217–218. 10.3163/1536-5050.103.4.013 26512226 PMC4613387

[B11] Cui Z. Luo F. Wang J. Diao J. Pan Y. (2023). Bibliometric and visual analysis of Kawasaki disease in children from 2012 to 2022. Front. Pediatr. 11, 1142065. 10.3389/fped.2023.1142065 37576134 PMC10413569

[B12] Daina A. Michielin O. Zoete V. (2019). SwissTargetPrediction: Updated data and new features for efficient prediction of protein targets of small molecules. Nucleic Acids Res. 47, W357-W364–W364. 10.1093/nar/gkz382 31106366 PMC6602486

[B13] Diaz-Beltran L. Cano C. Wall D. P. Esteban F. J. (2013). Systems biology as a comparative approach to understand complex gene expression in neurological diseases. Behav. Sci. 3, 253–272. 10.3390/bs3020253 25379238 PMC4217627

[B14] Dumago M. P. Agas R. A. F. Jainar C. J. E. Yap E. T. Co L. B. A. Ortin T. T. S. (2023). Stereotactic body radiation therapy with or without transarterial chemoembolization versus transarterial chemoembolization alone in early-stage hepatocellular carcinoma: A systematic review and meta-analysis. J. Gastrointest. Cancer. 10.1007/s12029-023-00940-5 37306936

[B15] Eyre-Walker A. Stoletzki N. (2013). The assessment of science: The relative merits of post-publication review, the impact factor, and the number of citations. PLOS Biol. 11, e1001675. 10.1371/journal.pbio.1001675 24115908 PMC3792863

[B16] Guo D. Jin J. Liu J. Ren M. He Y. (2023). Network pharmacological study of compound kushen injection in esophageal cancer. Curr. Comput.-Aided Drug Des. 19, 367–381. 10.2174/1573409919666230111155954 36635923

[B17] Hao D. C. Xiao P. G. (2014). Network pharmacology: A rosetta stone for traditional Chinese medicine. Drug Dev. Res. 75, 299–312. 10.1002/ddr.21214 25160070

[B18] Hassan-Montero Y. Guerrero-Bote V. P. De-Moya-Anegon F. (2014). Graphical interface of the <i&gt;SCImago journal and country rank</i&gt;: an interactive approach to accessing bibliometric information. Prof. Inf. 23, 272–278. 10.3145/epi.2014.may.07

[B19] Hirsch J. E. (2005). An index to quantify an individual’s scientific research output. Proc. Natl. Acad. Sci. U. S. A. 102, 16569–16572. 10.1073/pnas.0507655102 16275915 PMC1283832

[B20] Hu Y. Zhai W. Tan D. Chen H. Zhang G. Tan X. (2023). Uncovering the effects and molecular mechanism of Astragalus membranaceus (Fisch.) Bunge and its bioactive ingredients formononetin and calycosin against colon cancer: An integrated approach based on network pharmacology analysis coupled with experimental validation and molecular docking. Front. Pharmacol. 14, 1111912. 10.3389/fphar.2023.1111912 36755950 PMC9899812

[B21] Isaksson J. Berglund A. Louie K. Willén L. Hamidian A. Edsjö A. (2023). KRAS G12C mutant non-small cell lung cancer linked to female sex and high risk of CNS metastasis: Population-based demographics and survival data from the national Swedish lung cancer registry. Clin. Lung Cancer S1525-7304 (23), 507–518. –7. 10.1016/j.cllc.2023.05.002 37296038

[B22] Jiang M. Liu J. Li Q. Xu B. (2023). The trichotomy of HER2 expression confers new insights into the understanding and managing for breast cancer stratified by HER2 status. Int. J. Cancer 153, 1324–1336. 10.1002/ijc.34570 37314204

[B23] Jorgensen J. T. (2011). A challenging drug development process in the era of personalized medicine. Drug Discov. Today 16, 891–897. 10.1016/j.drudis.2011.09.010 21945860

[B24] Kanehisa M. Furumichi M. Tanabe M. Sato Y. Morishima K. (2017). Kegg: new perspectives on genomes, pathways, diseases and drugs. Nucleic Acids Res. 45, D353-D361–D361. 10.1093/nar/gkw1092 27899662 PMC5210567

[B25] Kim S. Thiessen P. A. Bolton E. E. Chen J. Fu G. Gindulyte A. (2016). PubChem substance and compound databases. Nucleic Acids Res. 44, D1202–D1213. 10.1093/nar/gkv951 26400175 PMC4702940

[B26] Kong Y. W. Dreaden E. C. Hammond P. T. Yaffe M. B. (2016). “Exploiting nanocarriers for combination cancer therapy,” in Intracellular delivery III fundamental biomedical technologies. Editors Prokop A. Weissig V. (Cham: Springer International Publishing), 375–402. 10.1007/978-3-319-43525-1_16

[B27] Li W. Huang T. Xu S. Che B. Yu Y. Zhang W. (2022). Molecular mechanism of tanshinone against prostate cancer. Molecules 27, 5594. 10.3390/molecules27175594 36080361 PMC9457553

[B28] Liu Y. Guo Z. Lang F. Li J. Jiang J. (2023). Anticancer effect of active component of Astragalus membranaceus combined with olaparib on ovarian cancer predicted by network-based pharmacology. Appl. Biochem. Biotechnol. 10.1007/s12010-023-04462-5 36976504

[B29] Lu J.-J. Pan W. Hu Y.-J. Wang Y.-T. (2012). Multi-target drugs: The trend of drug research and development. PLoS One 7, e40262. 10.1371/journal.pone.0040262 22768266 PMC3386979

[B30] Luo L. Chen Y. Ma Q. Huang Y. Hong T. Shu K. (2023). Exploring the mechanism of an active ingredient of ginger, dihydrocapsaicin, on triple negative breast cancer based on network pharmacology and *in vitro* experiments. Oncol. Lett. 25, 195. 10.3892/ol.2023.13781 37113393 PMC10126628

[B31] Luo X. Duan H. He L. (2020). A novel riccati equation grey model and its application in forecasting clean energy. Energy 205, 118085. 10.1016/j.energy.2020.118085 32546893 PMC7290234

[B32] Medical Subject Headings Home Page (2023). Medical subject Headings - Home page. Available at: https://www.nlm.nih.gov/mesh/meshhome.html (Accessed August 15, 2023).

[B33] Moon A. M. Kim H. P. Singal A. G. Owen D. Mendiratta-Lala M. Parikh N. D. (2023). Thermal ablation compared to stereotactic body radiation therapy for hepatocellular carcinoma: A multicenter retrospective comparative study. Hepatol. Commun. 7, e00184. 10.1097/HC9.0000000000000184 37314737 PMC10270501

[B34] Nelander S. Wang W. Nilsson B. She Q. Pratilas C. Rosen N. (2008). Models from experiments: combinatorial drug perturbations of cancer cells. Mol. Syst. Biol. 4, 216. 10.1038/msb.2008.53 18766176 PMC2564730

[B35] Peter M. (2020). Multi-targeting drugs: Past, present and future. Orvosi Hetil. 161, 523–531. 10.1556/650.2020.31703 32223419

[B36] Qayoom H. Alkhanani M. Almilaibary A. Alsagaby S. A. Mir M. A. (2023). A network pharmacology-based investigation of brugine reveals its multi-target molecular mechanism against Breast Cancer. Med. Oncol. 40, 202. 10.1007/s12032-023-02067-w 37308611

[B37] Ren Q. Zhang P. Zhang X. Feng Y. Li L. Lin H. (2023). A fibroblast-associated signature predicts prognosis and immunotherapy in esophageal squamous cell cancer. Front. Immunol. 14, 1199040. 10.3389/fimmu.2023.1199040 37313409 PMC10258351

[B38] Rosvall M. Bergstrom C. T. (2010). Mapping change in large networks. PLoS One 5, e8694. 10.1371/journal.pone.0008694 20111700 PMC2811724

[B39] Sheng P. Xie J. Wu Y. Xia X. Li B. Wu M. (2023). A network pharmacology approach for uncovering the mechanism of “kouchuangling” in radiation-induced oral mucositis treatment. Comb. Chem. High. Throughput Screen 26, 1042–1057. 10.2174/1386207325666220617151600 35718968

[B40] Sun X.-F. Singh S. P. (2023). Network pharmacology integrated molecular docking demonstrates the therapeutic mode of Panax ginseng against ovarian cancer. Trop. J. Pharm. Res. 22, 589–596. 10.4314/tjpr.v22i3.16

[B41] Szklarczyk D. Gable A. L. Lyon D. Junge A. Wyder S. Huerta-Cepas J. (2019). STRING v11: Protein-protein association networks with increased coverage, supporting functional discovery in genome-wide experimental datasets. Nucleic Acids Res. 47, D607-D613–D613. 10.1093/nar/gky1131 30476243 PMC6323986

[B42] Szklarczyk D. Morris J. H. Cook H. Kuhn M. Wyder S. Simonovic M. (2017). The STRING database in 2017: Quality-controlled protein–protein association networks, made broadly accessible. Nucleic Acids Res. 45, D362-D368–D368. 10.1093/nar/gkw937 27924014 PMC5210637

[B43] Szklarczyk D. Santos A. von Mering C. Jensen L. J. Bork P. Kuhn M. (2016). Stitch 5: augmenting protein–chemical interaction networks with tissue and affinity data. Nucleic Acids Res. 44, D380–D384. 10.1093/nar/gkv1277 26590256 PMC4702904

[B44] Tang H.-Z. Yang Z.-P. Lu S. Wang B. Wang Y.-Y. Sun X.-B. (2023). Network pharmacology-based analysis of heat clearing and detoxifying drug JC724 on the treatment of colorectal cancer. World J. Gastrointest. Oncol. 15, 90–101. 10.4251/wjgo.v15.i1.90 36684054 PMC9850754

[B45] Wang J. Dong P. Zheng S. Mai Y. Ding J. Pan P. (2023a). Advances in gut microbiome in metabonomics perspective: Based on bibliometrics methods and visualization analysis. Front. Cell. Infect. Microbiol. 13, 1196967. 10.3389/fcimb.2023.1196967 37325519 PMC10266355

[B46] Wang Y. Hu B. Feng S. Wang J. Zhang F. (2020). Target recognition and network pharmacology for revealing anti-diabetes mechanisms of natural product. J. Comput. Sci. 45, 101186. 10.1016/j.jocs.2020.101186

[B47] Wang Y. Wang B. Ma X. (2023b). A novel predictive model based on inflammatory response-related genes for predicting endometrial cancer prognosis and its experimental validation. Aging (Albany NY) 15, 4844–4860. 10.18632/aging.204767 37276865 PMC10292875

[B48] Wang Y. Zhang S. Li F. Zhou Y. Zhang Y. Wang Z. (2019). Therapeutic target database 2020: enriched resource for facilitating research and early development of targeted therapeutics. Nucleic Acids Res. 48, D1031-D1041. 10.1093/nar/gkz981 PMC7145558 31691823

[B49] Wei Y. Lin Y. Chen W. Liu S. Jin L. Huang D. (2021). Computational and *in vitro* analysis of plumbagin’s molecular mechanism for the treatment of hepatocellular carcinoma. Front. Pharmacol. 12, 594833. 10.3389/fphar.2021.594833 33912033 PMC8072012

[B50] Wu B. Lan X. Chen X. Wu Q. Yang Y. Wang Y. (2023). Researching the molecular mechanisms of taohong siwu decoction in the treatment of varicocele-associated male infertility using network pharmacology and molecular docking: A review. Med. Baltim. 102, e34476. 10.1097/MD.0000000000034476 PMC10402989 37543801

[B51] Wu C. Huang Z.-H. Meng Z.-Q. Fan X.-T. Lu S. Tan Y.-Y. (2021). A network pharmacology approach to reveal the pharmacological targets and biological mechanism of compound kushen injection for treating pancreatic cancer based on WGCNA and *in vitro* experiment validation. Chin. Med. 16, 121. 10.1186/s13020-021-00534-y 34809653 PMC8607619

[B53] Wu J. Luo D. Li S. (2020). Network pharmacology-oriented identification of key proteins and signaling pathways targeted by xihuang pill in the treatment of breast cancer. Breast Cancer-Targets Ther. 12, 267–277. 10.2147/BCTT.S284076 PMC7733446 33324095

[B54] Wu Z. Cheng K. Shen Z. Lu Y. Wang H. Wang G. (2022). Mapping knowledge landscapes and emerging trends of sonodynamic therapy: A bibliometric and visualized study. Front. Pharmacol. 13, 1048211. 10.3389/fphar.2022.1048211 36699067 PMC9868186

[B55] Xin W. Zi-Yi W. Jia-Hui Z. Shao L. (2021). TCM network pharmacology: A new trend towards combining computational, experimental and clinical approaches. Chin. J. Nat. Med. 19, 1–11. 10.1016/S1875-5364(21)60001-8 33516447

[B56] Xiong X. Song Q. Jing M. Yan W. (2023). Identification of PANoptosis-based prognostic signature for predicting efficacy of immunotherapy and chemotherapy in hepatocellular carcinoma. Genet. Res. (Camb) 2023, 6879022. 10.1155/2023/6879022 37313428 PMC10260314

[B57] Xu X. Feng C. (2021). Mapping the knowledge domain of the evolution of emergy theory: a bibliometric approach. Environ. Sci. Pollut. Res. 28, 43114–43142. 10.1007/s11356-021-14959-3 34152539

[B58] Yadav S. Gulati N. Jain A. Shetty D. C. (2023). Case series of head-and-neck adenosquamous carcinoma: Role of histogenetic model in immunotherapy as a future perspective for nonconventional squamous cell carcinoma. J. Cancer Res. Ther. 19, 505–510. 10.4103/jcrt.jcrt_2058_21 37313926

[B59] Yan Y.-C. Xu Z.-H. Wang J. Yu W.-B. (2022). Uncovering the pharmacology of Ginkgo biloba folium in the cell-type-specific targets of Parkinson’s disease. Front. Pharmacol. 13, 1007556. 10.3389/fphar.2022.1007556 36249800 PMC9556873

[B60] Zhang L. Ling J. Lin M. (2022). Artificial intelligence in renewable energy: A comprehensive bibliometric analysis. Energy Rep. 8, 14072–14088. 10.1016/j.egyr.2022.10.347

[B61] Zhang L. Ling J. Lin M. (2023). Carbon neutrality: a comprehensive bibliometric analysis. Environ. Sci. Pollut. R. 30, 45498–45514. 10.1007/s11356-023-25797-w 36800084

[B62] Zhang Y. Yao Y. Fu Y. Yuan Z. Wu X. Wang T. (2021). Inhibition effect of oxyepiberberine isolated from Coptis chinensis franch. On non-small cell lung cancer based on a network pharmacology approach and experimental validation. J. Ethnopharmacol. 278, 114267. 10.1016/j.jep.2021.114267 34087401

[B63] Zhong M. Lin M. (2022). Bibliometric analysis for economy in COVID-19 pandemic. Heliyon 8, e10757. 10.1016/j.heliyon.2022.e10757 36185135 PMC9509534

[B64] Zhou Z. Chen B. Chen S. Lin M. Chen Y. Jin S. (2020). Applications of network pharmacology in traditional Chinese medicine research. Evidence-Based Complementary Altern. Med. 2020, 1646905–1646907. 10.1155/2020/1646905 PMC7042531 32148533

