# Supplementary material for: Analyzing the research landscape: Mapping frontiers and hot spots in anti-cancer research using bibliometric analysis and research network pharmacology
Source: Front Pharmacol. 2023 Sep 7;14:1256188. doi: 10.3389/fphar.2023.1256188 (PMC10512719; doi:10.3389/fphar.2023.1256188)

Supplementary material

Contents

Supplementary information on software..2
Supplementary Table 1. GM(1,1) Model Forecasts Promising Surge in Publication Counts: Analysis of WoSCC and PubMed. 4
Supplementary Table 2. Publication contributions of top 30 countries. 5
Supplementary Table 3. Top 10 Most Cited Authors 6
Supplementary Table 4. The details of the top 11 clustered networks of co-cited references from 2008 to 2023 7
Supplementary Figure 1. Visualization of the Keyword Clustering Timeline from 2008 to 2023. 8
Supplementary Figure 2. Top 25 cited references with citation bursts. Red lines indicate the duration of the corresponding high citation period. 9
Supplementary Figure 3. Top 25 cited references with citation bursts. Red lines indicate the duration of the corresponding high citation period. 10

Supplementary information on software

The present study utilizes scientific bibliometrics analysis by employing CiteSpace, VOSviewer, R programming (Bibliometrix), and Scimago Graphica software to map out the literature on network pharmacology for anti-cancer research. This methodology produces a systematic mapping of research trends and impact networks in this field.

1. CiteSpace (6.1.R6)

CiteSpace is specialized software for scientific literature analysis that primarily aims to identify and demonstrate future scientific developments and trends. It utilizes cited literature and references to extract information such as title, abstract, author keywords (DE), and keywords plus (ID) to aid in co-authorship, co-occurrence, and co-citation analyses. CiteSpace has become increasingly sophisticated in visualizing co-citation analyses, providing science researchers with a convenient and intuitive analytical tool.

CiteSpace offers three forms of visualization, including clustering visualization, time interval visualization, and timeline visualization. Clustering visualization primarily reveals the formation of different thematic structures in the field, while time interval visualization presents the evolutionary trends of each theme over time. Timeline visualization conveniently illustrates the period involved in each research topic. This study employs the three visualizations mentioned above while integrating the analysis of keyword occurrences to investigate the research trends and hotspots in network pharmacology for anti-tumor purposes.

2. VOSviewer (1.6.19)

VOSviewer is a software tool for constructing optical networks. Compared with CiteSpace, it is more convenient regarding basic literature information statistics. Therefore, this study primarily employs CiteSpace for visualization, while VOSviewer complements the results. The main contribution of VOSviewer lies in its analysis of international collaboration networks.

It is important to note that noun abbreviations must be standardized and national boundaries clearly defined in this study. For instance, Wales, England, Scotland, and Northern Ireland should be uniformly summarized as the United Kingdom. Similarly, Taiwan should be summarized as part of China.

3. Scimago Graphica (Beta 1.0.35)

Scimago Graphica is a scientific collaboration network visualization software that can transform scientific collaboration networks into graphics for better analysis and understanding of scientific collaboration relationships. It has an intuitive user interface and simple operation, allowing users to easily import data, set node and edge properties, select layout algorithms, etc. Additionally, it has advanced visualization features, such as node and edge color, shape, size, label settings, as well as node and edge filtering,

clustering, annotation and other functions. Scimago Graphica supports multiple data formats, such as CSV, XLSX, GraphML, GEXF, GML, and can be used in conjunction with other software, such as VosViewer, Gephi, etc. In this study, we used this tool to visualize the national collaboration network and analyze the international cooperation status in the field of network pharmacology and anti-tumor.

Supplementary Table 1. GM(1,1) Model Forecasts Promising Surge in Publication Counts: Analysis of WoSCC and PubMed.

Year	WoS counts	Stimulation	pubmed counts	Stimulation
2008	2	0.350087	1	0.250077
2009	2	0.610852	1	0.411281
2010	4	1.06585	2	0.676399
2011	6	1.859758	2	1.112415
2012	12	3.245014	9	1.829495
2013	21	5.662088	12	3.008814
2014	27	9.879541	13	4.94834
2015	29	17.2384	16	8.138113
2016	49	30.07856	21	13.38406
2017	51	52.48282	16	15.66766
2018	96	91.57507	25	28.08311
2019	148	159.7855	46	50.33687
2020	265	278.8031	73	90.22508
2021	506	486.4718	188	161.7217
2022	774	783.3616	287	289.8741
2023	222	1312.157	95	519.5777
2024	-	2197.907	-	931.3041
2025	-	3681.568	-	1669.293
2026	-	6166.749	-	2992.083

Supplementary Table 2. Publication contributions of top 30 countries.

Country	Publication	Citation	Average Citation	Cluster
China	1765	13823	7.8317	1
USA	254	5764	22.6929	2
India	67	277	4.1343	1
South Korea	61	333	5.459	1
UK	58	3338	57.5517	2
Germany	41	757	18.4634	2
Japan	23	153	6.6522	2
Saudi Arabia	21	40	1.9048	1
Egypt	19	302	15.8947	1
France	18	261	14.5	1
Canada	15	67	4.4667	3
Australia	13	173	13.3077	3
Italy	13	211	16.2308	1
Pakistan	13	52	4	1
Netherlands	12	410	34.1667	2
Bangladesh	11	39	3.5455	1
Finland	10	471	47.1	3
Singapore	9	594	66	3
Spain	9	297	33	2
Sweden	9	372	41.3333	2
Switzerland	9	51	5.6667	2
Malaysia	8	52	6.5	1
Russia	8	163	20.375	2
Thailand	7	26	3.7143	1
Iran	6	61	10.1667	3
Mexico	6	47	7.8333	3
Turkey	6	58	9.6667	1
Brazil	5	267	53.4	2
Ireland	5	167	33.4	2
United Arab Emirates	5	113	22.6	1

Supplementary Table 3. Top 10 Most Cited Authors

Author	Publication	Citation	Average Citation
Zhang Ying	17	548	32.24
Popel Aleksander S.	17	482	28.35
Feng Yibin	10	396	39.60
Wang Ning	10	396	39.60
Wang Yonghua	19	388	20.42
Li Shao	7	363	51.86
Li Rong	14	359	25.64
Gu Jiangyong	5	345	69.00
Cheng Feixiong	8	326	40.75
Li Yan	17	324	19.06

Supplementary Table 4. The details of the top 11 clustered networks of co-cited references from 2008 to 2023

Cluter ID	Size	Silhouette	Mean (Year)	Top terms (log-likelihood ratio, p-level)	Representative reference
0	219	0.743	2019	molecular docking (1030.48, 1.0E-4); molecular docking analysis (961.48, 1.0E-4); colon cancer (815.87, 1.0E-4); endometrial cancer (794.75, 1.0E-4); potential mechanism (758.61, 1.0E-4)	Zheng, Y (2022.0) Active ingredients and molecular targets of taraxacum mongolicum against hepatocellular carcinoma: network pharmacology, molecular docking, and molecular dynamics simulation analysis. PEERJ, V10, P29 DOI 10.7717/peerj.13737
1	103	0.781	2015	molecular docking (1533.23, 1.0E-4); network pharmacology approach (1466.05, 1.0E-4); compound kushen injection (1246.87, 1.0E-4); hedyotis diffusa willd (903.1, 1.0E-4); gastric precancerous lesion (804.65, 1.0E-4)	Fang, J (2018.0) In silico polypharmacology of natural products. BRIEFINGS IN BIOINFORMATICS, V19, P19 DOI 10.1093/bib/bbx045
2	92	0.92	2010	multi-target anticancer therapy (246.14, 1.0E-4); drug discovery (235.18, 1.0E-4); vitexicarpin act (234.93, 1.0E-4); novel angiogenesis inhibitor (234.93, 1.0E-4); computational model (223.86, 1.0E-4)	Tang, J (2014.0) Network pharmacology strategies toward multi-target anticancer therapies: from computational models to experimental design principles. CURRENT PHARMACEUTICAL DESIGN, V20, P14 DOI 10.2174/13816128113199990470
3	63	0.97	2017	herbal drug fdy003 (861.01, 1.0E-4); compound kushen injection (700.73, 1.0E-4); herbal drug fdy2004 (659.93, 1.0E-4); ulcerative colitis (594.23, 1.0E-4); breast cancer treatment (590.93, 1.0E-4)	Lee, H (2021.0) A network pharmacology analysis of the systems-perspective anticancer mechanisms of the herbal drug fdy2004 for breast cancer. NATURAL PRODUCT COMMUNICATIONS, V16, P16 DOI 10.1177/1934578X211049133
4	44	0.855	2012	using systems pharmacology (173.66, 1.0E-4); resource review (160.23, 1.0E-4); pancreatic ductal adenocarcinoma therapy (160.23, 1.0E-4); oncology drug development (158.24, 1.0E-4); rectifying cancer drug discovery (146.84, 1.0E-4)	Kirouac, DC (2016.0) Using systems pharmacology to advance oncology drug development. SYSTEMS PHARMACOLOGY AND PHARMACODYNAMICS, V23, P43 DOI 10.1007/978-3-319-44534-2_19
5	43	0.999	2007	next paradigm (75.59, 1.0E-4); drug discovery (56.25, 1.0E-4); systems pharmacology (52.66, 1.0E-4); annual meeting symposium (50.46, 1.0E-4); critical path initiative (50.46, 1.0E-4)	Hopkins, AL (2008.0) Network pharmacology: the next paradigm in drug discovery. NATURE CHEMICAL BIOLOGY DOI 10.1038/nchembio.118
6	42	0.896	2018	bioinformatics investigation (652.18, 1.0E-4); bioactive constituent (548.99, 1.0E-4); predicting therapy target (500.87, 1.0E-4); tripterygium wilfordii (439.04, 1.0E-4); comprehensive application (377.21, 1.0E-4)	Jiao, X (2021.0) A comprehensive application: molecular docking and network pharmacology for the prediction of bioactive constituents and elucidation of mechanisms of action in component-based chinese medicine. COMPUTATIONAL BIOLOGY AND CHEMISTRY DOI 10.1016/j.compbiolchem.2020.107402
7	39	0.919	2014	natural product (634.57, 1.0E-4); precision oncology (402.76, 1.0E-4); systems pharmacology (240.15, 1.0E-4); drug-target interaction (230.69, 1.0E-4); new targeted cancer therapy (230.69, 1.0E-4)	Fang, J (2017.0) Quantitative and systems pharmacology. 1. in silico prediction of drug-target interactions of natural products enables new targeted cancer therapy. JOURNAL OF CHEMICAL INFORMATION AND MODELING, V57, P15 DOI 10.1021/acs.jcim.7b00216
8	35	0.986	2018	t cell engager (348.85, 1.0E-4); quantitative systems pharmacology model (296.84, 1.0E-4); solid tumor (283.85, 1.0E-4); antitumor potency (167.33, 1.0E-4); combination therapy (167.33, 1.0E-4)	Ma, H (2020.0) Combination therapy with t cell engager and pd-l1 blockade enhances the antitumor potency of t cells as predicted by a qsp model. JOURNAL FOR IMMUNOTHERAPY OF CANCER DOI 10.1136/jitc-2020-001141
9	25	0.991	2019	therapeutic target (235.1, 1.0E-4); exploring anti-liver cancer target (223.19, 1.0E-4); pharmacological characteristics (212.01, 1.0E-4); uterine corpus (212.01, 1.0E-4); endometrial carcinoma patient (212.01, 1.0E-4)	Zhao, F (2021.0) Exploring anti-liver cancer targets and mechanisms of oxyresveratrol: in silico and verified findings. BIOENGINEERED, V12, P10 DOI 10.1080/21655979.2021.1985328
10	22	0.975	2012	quantitative systems pharmacology perspective (110.38, 1.0E-4); pharmacology approaches (94.52, 1.0E-4); antiangiogenic therapy (94.52, 1.0E-4); case study (85.39, 1.0E-4); signaling network (78.68, 1.0E-4)	Klinke, DJ (2015.0) Enhancing the discovery and development of immunotherapies for cancer using quantitative and systems pharmacology: interleukin-12 as a case study. JOURNAL FOR IMMUNOTHERAPY OF CANCER DOI 10.1186/s40425-015-0069-x

Supplementary Figure 1. Visualization of the Keyword Clustering Timeline from 2008 to 2023.

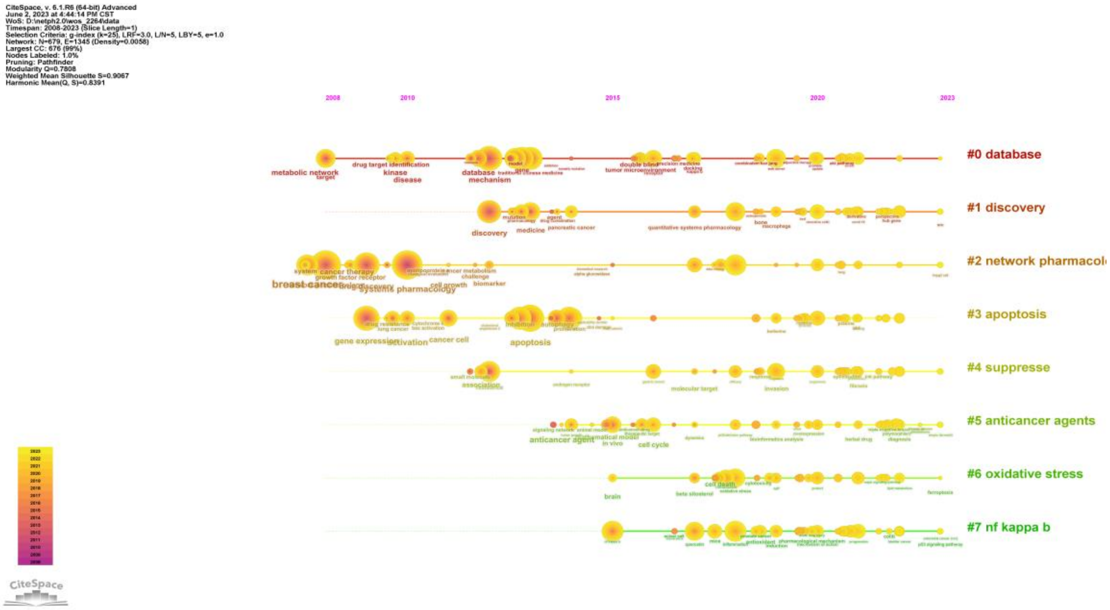

Supplementary Figure 2. Top 25 cited references with citation bursts. Red lines indicate the duration of the corresponding high citation period.

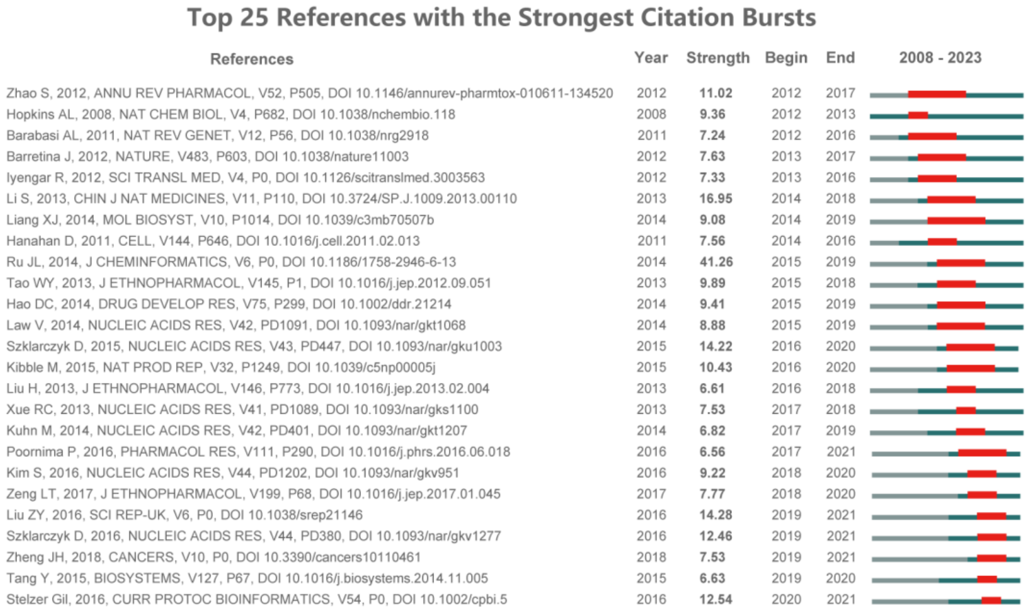

Supplementary Figure 3. Top 4 centrality research institutions in NPART.

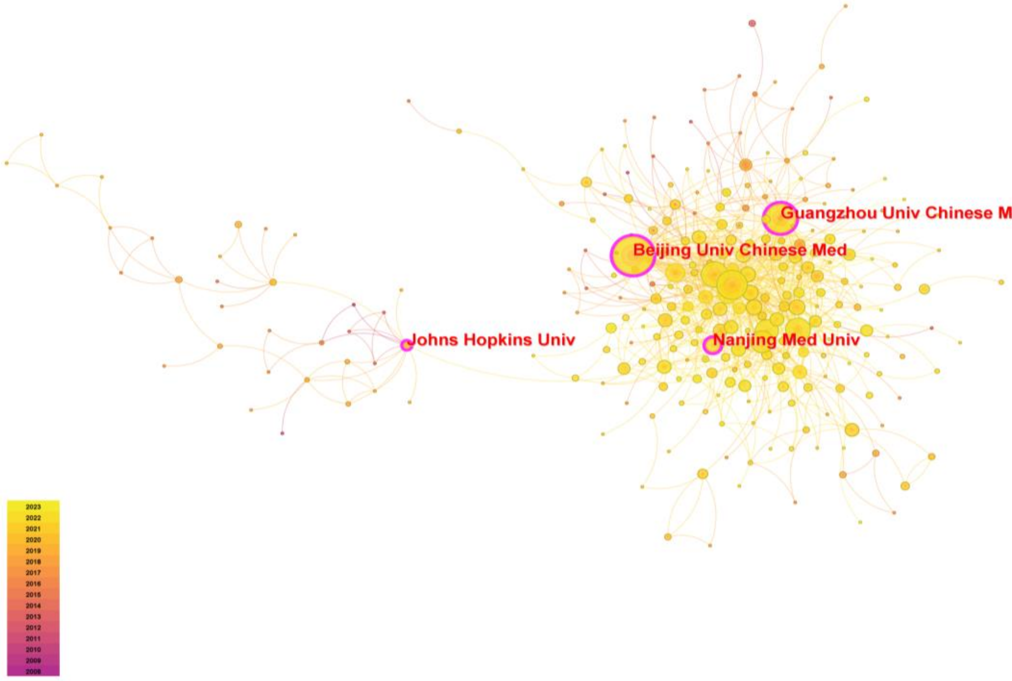

Supplement: Supplementary file 1 [file DataSheet1.PDF]
